# Explainable Model Fusion for Customer Journey Mapping

**DOI:** 10.3389/frai.2022.824197

**Published:** 2022-05-11

**Authors:** Kotaro Okazaki, Katsumi Inoue

**Affiliations:** ^1^Department of Informatics, School of Multidisciplinary Sciences, The Graduate University for Advanced Studies, SOKENDAI, Tokyo, Japan; ^2^SONAR Inc., Tokyo, Japan; ^3^Principles of Informatics Research Division, National Institute of Informatics, Tokyo, Japan

**Keywords:** XAI, marketing, customer journey mapping, process mining, generative model, Boolean network, LFIT, over-the-top media services

## Abstract

Due to advances in computing power and internet technology, various industrial sectors are adopting IT infrastructure and artificial intelligence (AI) technologies. Recently, data-driven predictions have attracted interest in high-stakes decision-making. Despite this, advanced AI methods are less often used for such tasks. This is because AI technology is a black box for the social systems it is meant to support; trustworthiness and fairness have not yet been established. Meanwhile in the field of marketing, strategic decision-making is a high-stakes problem that has a significant impact on business trends. For global marketing, with its diverse cultures and market environments, future decision-making is likely to focus on building consensus on the formulation of the problem itself rather than on solutions for achieving the goal. There are two important and conflicting facts: the fact that the core of domestic strategic decision-making comes down to the formulation of the problem itself, and the fact that it is difficult to realize AI technology that can achieve problem formulation. How can we resolve this difficulty with current technology? This is the main challenge for the realization of high-level human-AI systems in the marketing field. Thus, we propose customer journey mapping (CJM) automation through model-level data fusion, a process for the practical problem formulation known as explainable alignment. Using domain-specific requirements and observations as inputs, the system automatically outputs a CJM. Explainable alignment corresponds with both human and AI perspectives and in formulating the problem, thereby improving strategic decision-making in marketing. Following preprocessing to make latent variables and their dynamics transparent with latent Dirichlet allocation and a variational autoencoder, a *post-hoc* explanation is implemented in which a hidden Markov model and learning from an interpretation transition are combined with a long short-term memory architecture that learns sequential data between touchpoints for extracting attitude rules for CJM. Finally, we realize the application of human-AI systems to strategic decision-making in marketing with actual logs in over-the-top media services, in which the dynamic behavior of customers for CJM can be automatically extracted.

## Introduction

Thanks to advances in computing power and internet technology, various industrial sectors are adopting IT infrastructure and artificial intelligence (AI). Recently, data-driven predictions have attracted interest in high-stakes decision-making (Veale et al., 2018), yet advanced black-box methods are less often used (Rudin, 2019). Our research aims to apply explainable AI technology to the strategic decision-making in the field of marketing. For a company, marketing (Kotler, 1972) globally encompasses all business activities aimed at creating demand for the value that it offers.

In the field of marketing, strategic decision-making is a high-stakes problem that has a significant impact on business trends. However, for global marketing, with its diverse cultures and market environments, future decision-making is likely to focus on building consensus on the formulation of the problem itself rather than on solutions for achieving the goal (Conrad, 2019). This is because the diversity of the environment is created from different perspectives and degrees of literacy (Quade and Grace, 1989). For this reason, black-box methods are hard to apply to strategic decision-making, which is a human-based judgement process. This is because AI technology is a black box for the social systems it is meant to support; trustworthiness and fairness have not yet been established (Miller, 2019). Data-driven predictions using AI in marketing are limited to the operational level (Whittle et al., 2019). The challenge is limited both in optimizing decisions and in improving efficiency under known and highly repeatable situations (Johnson et al., 2019). The study of intelligence for strategic decision-making belongs to the domain of artificial general intelligence (AGI) (Baum, 2017), which aims to discover unseen tasks and to modify tasks based on a given situation.

The difficulty of realizing AGI can be thought of as building a technology that can formulate problems from scratch (Duch et al., 2008). There is a gap between two important facts: the fact that the core of domestic strategic decision-making comes down to the formulation of the problem itself, and the fact that it is difficult to realize AI technology that can achieve problem formulation. How can we fill this gap with current technology? This is the main challenge for the realization of high-level human-AI systems in the marketing field. This raises three questions. First, what can replace the problem formulation in marketing strategy? Second, what is the kind of process that can be derived from the result of problem formulation? Finally, can the process be functional in a human-AI system that contributes to strategic marketing decisions?

To answer the first question, we claim that customer journey mapping (CJM) (Lemon and Verhoef, 2016) is close to a formulation of the marketing problem itself. CJM is one of the most important analyses that can overlay marketing strategy goals and customer behavior by drawing customer journeys. CJM represents the flow of customer experiences, a dynamic process akin to a “journey” of universal purchasing attitudes. Visualizing the customer journey deepens understanding of the dynamics and feedback of the important information and is one essential process in a customer-facing business, whether as a reactive measure or a proactive control (Rosenbaum et al., 2017). The problem formulation in strategic decision-making defines the subject of the problem and its model. The elements of the problem are expressed as manipulable variables and non-manipulable constants, and the relationships and structures among these elements that prevent the subject from transitioning to the target state are represented (Bell et al., 1988). In fact, the customer journey has involved the necessary elements for formulating strategic decision-making problems in social systems. First, the journey has a subject. The subject in the customer journey is the customer that is conducting buying behavior. Next, the touchpoints are symbols that represent the variables and elements that can and cannot be manipulated as nodes. The nodes are connected by edges that indicate the behavioral process. In other words, it is an expression of the relationship between variables and elements. The customer journey is a directed graph showing customer behavior as well as representing the customer's state as a transition to the goal of purchasing. Moreover, since the model includes a goal, it is important in decision-making. Because of these characteristics, the customer journey is used practically in marketing strategy decisions. Therefore, CJM is synonymous with the formulation of the problem itself. On the other hand, the current CJM has some shortcomings that should be improved. Since the modeling of consumer behavior is always provisional, the essence of strategic decision-making in marketing is in the consensus between the decision-makers' beliefs and contexts (Benzarti et al., 2021). There is always a conflict between quantitative efficiency management and qualitative structural understanding. In some cases, the journeys are drawn with the purpose of reflecting the gathered data, but they are rarely designed quantitatively in a data-driven manner beforehand. Also, in creating such models, it is hard to avoid the subjective influence of the decision maker. Therefore, the current CJM is vulnerable to interpolation and extrapolation facts (Richardson, 2010). For example, it is difficult to represent paths that do not lead to the target state, or in other words, to represent the true issues in the journey. However, these shortcomings can be easily solved from the perspective of AI systems. The techniques for automating CJM are, in short, a combination of finding the variables in the model, visualizing the structure and knowledge among them, and maintaining the prediction accuracy of the model by adjusting the parameters. These technologies are already well established and have achieved success in each individual task (Jordan and Mitchell, 2015). If we can combine the models appropriately in an explainable artificial intelligence (XAI) framework (Adadi and Berrada, 2018), it will be possible to both visualize the solution process and achieve accuracy. The problem formulation is whether we can apply AI technology for CJM automation for conducting the social decision-making process. The automation of CJM is a real-world application of AI technology that incorporates a high-level human decision-making process with explainable, understandable, and accurate functionality.

For the second question, we propose CJM automation through model-level data fusion (Diez-Olivan et al., 2019), a process called explainable alignment. Using domain-specific requirements and observations as inputs, the system automatically outputs a CJM. Explainable alignment corresponds with human and AI perspectives and in facilitating an unknown world view, thereby improving the original observation environment. This alignment creates four layers while building explainability in an overall feedback loop: (1) an algorithmic framework layer, (2) a model fusion layer incorporating social system and AI system perspectives, (3) an automatic CJM output and knowledge extraction layer, and (4) a data fusion space layer. Please refer to Chapter 4 for details. For a social system, we incorporate process mining (Van Der Aalst et al., 2011) that is linked to specific sequentially recorded business actions. Illuminating the event logs from the customer behavior environment with process mining—a process that was originally used to improve internal processes—ensures social consensus in technology selection. For an AI system, we employ research into generative models (Jebara, 2012) with inductive logic programming (ILP) (Muggleton, 1991) to achieve appropriate model fusion, which is comparable to process mining. Generative models and ILP are one of the most active research areas in the field of AI inference technology and are now being widely used in everything, for example, data augmentation to investigate unknown infectious diseases by the former (Waheed et al., 2020) and advanced satisfiability problem solving for autonomous driving controls by the latter (Suchan et al., 2019). The customer journey, automatically output as the third layer, is both an explanation of the environment learned in the black box and a predictor that is continuously updated as a behavioral model. Finally, in the fourth layer, data fusion (Arrieta et al., 2020) for observation, it is necessary to be able to accurately capture and verify how the customer responds to the measures. The alignment toward these four interacting layers supports fair and transparent decision-making while fostering literacy appropriate to the decision makers. The observation results are processed as follows, according to the interpretable framework in which the modeling was established. First, a long short-term memory (LSTM) (Murdoch and Szlam, 2017) is trained on the observed viewing logs, and then preprocessing is applied to generate the oracle for all episodes for each viewer by assimilating real data with predictions. We acquire both *post-hoc* explainability (Arrieta et al., 2020) and CJM automation with regards to this black-box method through the following three stages. The first is to visualize static latent features with a latent Dirichlet allocation (LDA) (Blei et al., 2003) and dynamic features with a variational autoencoder (VAE) (Kingma and Welling, 2013). This stage corresponds to discovery in process mining and dimensional reduction. The next stage is to use *post-hoc* explainability (Arrieta et al., 2020) to identify which features are necessary to focus on. Only the viewing logs affected by the feature through a learned model are resampled. This stage corresponds to conformance checking in process mining and feature optimization. A hidden Markov model (HMM, a *post-hoc* explainability contrasting to LSTM) (Baum and Petrie, 1966) is learned from that log. The state transition diagram becomes the customer journey. Furthermore, a set of rules is extracted by applying the learning from interpretation transition (LFIT) (Inoue et al., 2014) as an ILP process. This stage comprises enhancement from a process mining prospective as well as a knowledge update in the AI system.

For the last question, a practical application of human-AI systems to strategic decision-making in marketing is realized. This paper proposes this application with actual customer behavior logs in over-the-top (OTT) media services (Moro-Visconti, 2021) as the extension of our previous work (Okazaki and Inoue, 2017). This provides content that was formerly delivered over different platforms to a range of devices via online streaming technology. Revenue structures vary widely across advertising media and subscription services, but all have one thing in common: a business can be established only with content that keeps viewers constantly engaged (Moro-Visconti, 2021). In such services, content viewing can be captured as immediate data. Programming, production, and promotion are already based to some extent on data-driven decision-making (Moro-Visconti, 2021). This trend toward a business structure that combines online infrastructure and digital devices is expanding to all marketing domains, not just entertainment content (Turkanik and Johnson, 2020). Therefore, applying our research to the OTT media business is equivalent to approaching the possibility of a human–AI collaboration system in a more realistic marketing environment. We analyze viewing-related behaviors, which are an extremely popular form of OTT content. Given the observation inherent in serial dramas, we determine which latent variables to focus on from both process mining and generative models with ILP perspectives, output the customer journeys from logs containing such properties, and achieve model fusion that transforms them into behavioral rules. Section Related Works of this paper introduces trends in related works and demonstrates the academic originality of our research. Section Concepts and Background introduces the necessary basic concepts and scope with the flow of explainable alignment as well as details of the four layers that it explains together. Section Method and Implementation explains the method and implementation applied to the actual viewing logs from the OTT media service. Section Experiment and Results discusses an experiment using broadcast logs from a serial drama and its results. Finally, Section Conclusion discusses the conclusion and the direction of future research.

## Related Works

As research on the application of machine learning to decision-making progressed, it became clear that the output process for learning-based predictions could not be visualized (Castelvecchi, 2016)—this is the so-called “black-box” problem (Ribeiro et al., 2016). A movement to study AI explainability itself (Rudin, 2019), in an era where collaboration with humans is a must (Gunning and Aha, 2019), has begun and is presently ongoing. Various developments are underway, including establishing a taxonomy of AI system explainability (Arrieta et al., 2020), measurement methods (Hoffman et al., 2018), and meta-analysis (Gilpin et al., 2018). Our proposal applies to the decision-making in real business data environments (Loyola-Gonzalez, 2019).

In collaborative systems between humans and AI, Miller (2019) raises issues in terms of social–scientific properties that govern the decision-making process. Social engineering research is concerned mainly with improving business administration processes (O'Neill and Sohal, 1999). We aim at explainability laid out in terms of business process re-engineering. We employed process mining (Van Der Aalst et al., 2011; R'bigui and Cho, 2017) and presented the possibility of using it to complement machine learning, but our research delves into the framework and validation covering the entire process in an actual application study.

Learning and prediction from real-world data environments have become an important research area in applied science in general (Kalman, 1960). Predicting parameters in regression models from large-scale observation data (Rauch et al., 1965) has produced many results in meteorology. In sensing-based motion search (Patterson et al., 2003), various studies (Dong and Andrews, 2009) have aimed to recognize motion patterns using machine learning, including the control (Cook and Das, 2004) of integrated environments (Wang et al., 2005) as well as evacuation plans (Helbing et al., 2000). Our research is original because it approaches the extraction of the latent attitudes behind the behavior, despite the fact that the behavior itself is easily defined in terms of content viewing.

Although there have been previous attempts to use machine learning for TV broadcasting (Bennett and Lanning, 2007), most research has focused on recommendation algorithms. CJM (Richardson, 2010; Lemon and Verhoef, 2016) aimed at customer behavior typically proposes a consensus based on *ad hoc* marketing research (Halvorsrud et al., 2016), but only a few scattered studies have targeted automation (Rosenbaum et al., 2017). Meanwhile, Bernard and Andritsos (2017) proposed CJM from a process-mining perspective and did not reach the demonstration stage. The originality of our proposal lies in the use of real data and an approach to CJM, with informatics technology based on a management engineering framework, which facilitates both automation and explainability.

In the field of natural language processing, state-space models (Aoki, 2013) are used mainly when latent variables are included in the model, and parameter estimation methods from Markov chains (Astrom, 1965) and Bayesian hierarchical models (Murphy, 2002) are used and routinely implemented in speech recognition (Zweig and Russell, 1998). The technique of estimating Bayesian networks between events with conditional probabilities has been used as a statistical causal search technique in sociology (Pearl, 2009). From the field of deep learning, it is becoming common to visualize the hidden layers of unsupervised learning with autoencoders and generative adversarial networks (Raiko and Tornio, 2009; Kingma and Welling, 2013) and recurrent neural networks (RNNs) that have been trained over a long period with *post hoc* analysis (Murdoch and Szlam, 2017), as well as visualizing the attention of transformer-based pre-training models for transfer learning (Vig, 2019). These techniques, which can simultaneously extract latent variables and reduce observation dimensionality, have been used in places in this paper.

In the life sciences and bioinformatics, a wide range of research (Liang et al., 1998; Akutsu et al., 1999; Murphy and Mian, 1999) has extracted the interaction mechanisms between chemicals involved in genetic expression. These have evolved into probabilistic Boolean networks (BNs) (Shmulevich et al., 2002) and methods for estimating dynamic Bayesian networks (Lähdesmäki et al., 2006), but as the number of variables increases, the challenge becomes in how to address computational complexity (Louizos et al., 2017; Shi et al., 2019). In contrast, deductive algorithms, which solve the satisfiability problem from time-series observations, are a promising solution. In natural language processing, this technique can be applied to parsing analysis of sentences as well as speech intent recognition (Sato and Kameya, 1997). In logic programming (Pearl, 1988; Muggleton, 1991; Poole, 1993), this technique enables inference from uncertain partial observations using probabilistic logic formulas (Friedman et al., 2000; Raedt and Kersting, 2008). In summary, discrete environmental changes in which latent variables are acting dynamically can be regarded as a state-space model estimation (Durbin and Koopman, 2012).

Among these options, we use LFIT techniques (Inoue et al., 2014) developed from work showing the compatibility of deterministic BNs with logic programming (Inoue, 2011) for automating CJM as a Boolean network with perturbation (BNp) and with extracting the rules. LFIT (Inoue et al., 2014) has been further developed for computation in continuous space, including the time series of biological reactions (Ribeiro et al., 2017), behavior detection on TV viewing (Okazaki and Inoue, 2017), and rule extraction from deep learning networks (Gentet et al., 2016), demonstrating its applicability to big data and online learning (Sakama et al., 2021).

## Concept and Background

### Explainable Artificial Intelligence

Since the publication of DARPA's XAI study (Gunning and Aha, 2019), there have been several meta-analyses (Gilpin et al., 2018) evaluating subsequent recent trends. The taxonomy presented in Guidotti et al. (2018) categorizes the problem of opening black boxes itself as the object to be explained. The solution to any black-box problem is formulated as a special case of a general classification problem having the common goal of providing an interpretive and accurate predictive model. These solutions can be broadly divided into those that solve inverse problems and those that design explanations. Solving an inverse problem means to input a decision-making result from a black box as an observation and to output an explanation that arrives at that result. By contrast, designing explanations is called “transparent box design,” where decision-making results from a black box are input as a training example that should be learned alongside development of an explaining interpretable prediction model. In general, an explanation is an interface between a decision-maker and a human being regarding a made decision, with the terminology used in Arrieta et al. (2020). The terminology of explanations can be defined in the four quadrants created from the axes of activity–passivity and model understandability–human understandability provided by the explanation. Transparency is a passive model understandability that provides clarity of the functioning of the model itself. Generally, the simpler the deductive model is, the more transparent it is. Interpretability is a passive human understandability that provides meaning with conviction and value to a human observer. Comprehensibility, which is a term that indicates human understandability and activity, is the capacity for human-style expression of the knowledge learned by the model. Finally, explainability means active model understandability. It refers to any action or procedure intended to reveal in detail the model's internal functioning. The concept of explainability in this paper is based on the above.

How an AI system can be explained depends on whether the model can be translated from its design or by external XAI technology. The former is called a “transparent model,” and the latter is called “*post-hoc* explainability.” The explainability required for advanced human-AI systems is based on whether they are black-box machine learning structures or frameworks with *post-hoc* explainability that are involved as social decision-making processes that address real-world problems (Arrieta et al., 2020). Our strategy is based on the latter as a practical choice.

### Problem Formulation

The essence of the strategic problem in social systems (Quade and Grace, 1989) is how to allocate resources in a balanced manner to areas that compete temporally and spatially. Temporal conflicts are those that determine the allocation of effort to the future. Spatial conflicts are conflicts in determining the proportion of human, material, and financial resources to be allocated to various activities, including entry into new fields and exit from existing fields. Strategic decision-making is a series of processes for solving problems, such as researching ways to deal with these strategic problems and developing and implementing strategic plans. However, the future contains many uncertain factors, and it is necessary to justify the prior investment of resources, at the expense of the present to some extent, for the future. The allocation of resources to organizational activities also means the determination of priorities among activities, which in turn means changing the rules for the distribution of authority and benefits implicitly determined by the current form of activities. In addition, efforts and persuasion are needed to make the resulting interest structure acceptable. In this way, strategic decision-making involves a great deal of unavoidable chaos due to the friction caused by uncertainty and the spatial and temporal conflicts of interest. In order for an organization or system to remain adaptable under the changing social environment, it must have sufficient diversity within it at the same level as the environment itself (Ashby, 1991). This trend has become increasingly pronounced in recent years. This fundamental nature of strategic decision-making requires a process of coordination between different positions, contexts, and literacies. The first and most important step is to formulate the problem. The problem formulation involves examining the roots and background of the problem, identifying the causes that should be taken into account in the situation, and describing the relevant activities of the actors and influencers in the model, including the possible alternative paths to the goals and objectives that one wants to reach through the solution. An uncontrollable environment, such as complex customer behaviors, is assumed to be an explanatory variable in the model, especially in strategic decision-making in marketing. This is quite similar to problem formulation in marketing.

### Decision-Making in Social Systems

Miller (2019) provided insights on forms of XAI research from the perspective of social science. They discuss how people incorporate cognitive bias and social expectations in the process of explaining, citing a wide range of academic fields, including philosophy, psychology, and cognitive science. Explanations in social systems show four properties that are likely to arise while approaching consensus. First, explanations in society are contrastive. Based on local discoveries of an actual event, we try to approach more universal principles of the world surrounding these events by contrasting them with hypotheses about specific confounding factors. Second, social explanations make biased selections. Rather than seeking complete explanations for events and their actual causes, people skillfully extract a limited number of factors that they are comfortable with. However, such choices are often influenced by cognitive bias. Third, in social explanations, likelihood is less important than cause. In other words, using statistical generation to explain why an event occurs is considered inappropriate without a description of the causal relationships behind that generation. Fourth, explanations in society are contextual interactions. People transfer knowledge presented as parts of conversations and interactions but in relation to the beliefs of the explainer and the explainee. Although an event can have many causes, discussions of its explanation will often occur with explainees focusing only on details that are self-contextually convenient, while explainers select just one detail based on various evaluation criteria. As a whole, these properties suggest that the essence of social consensus in decision-making lies in a mutual facilitation for obtaining a worldview of which all decision makers agree and accept, even if doing so results in unexpected errors. Facilitation (Zajonc, 1965) is a responsible intervention in group processes that encourages the activation and cooperation of organizations and communities. As a result, the key to collaboration between human social systems and AI systems is to establish a comprehensive governance code for each domain.

### Customer Journey Mapping

**Figure 1:**
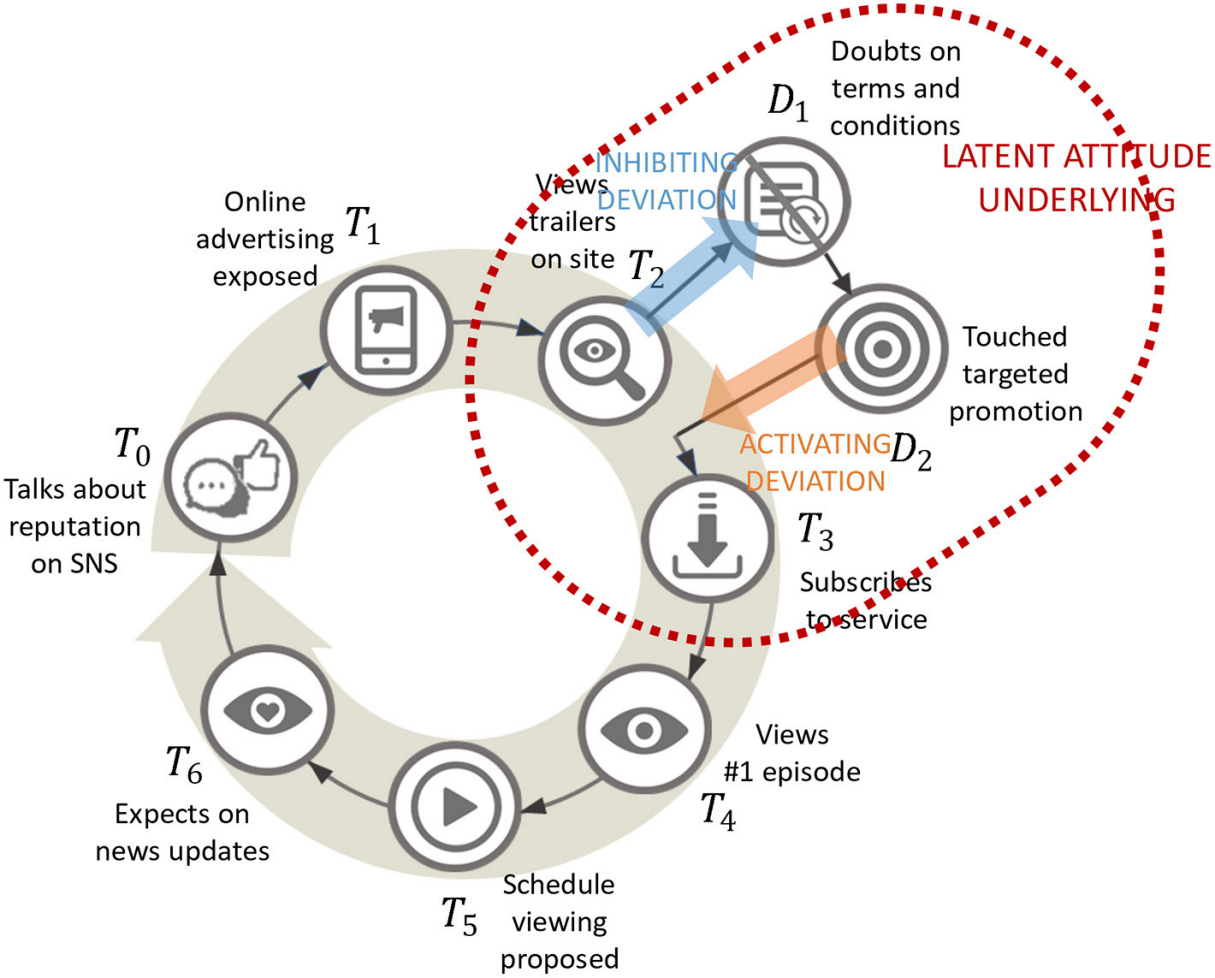
Customer journey mapping (CJM). An example of planned CJM based on content viewing.

Over the past half century, marketing aimed at creating lasting market demand has evolved from conceptual models for discussion to engineering models that can be applied in the real worlds (Lemon and Verhoef, 2016). Engagement through customer experience is increasingly considered the most important concept for management that augments brand value. Customer experience is defined as the multidimensional construction of a customer's cognitive, emotional, behavioral, sensory, and social responses to offers from a company or brand during the processes that lead to a purchase by that customer. CJM models the flow of this customer experience. As in Figure 1, CJM produces a segmented timeline showing the order of occurrence of events as phases in the customer experience, expressing them as funnels or loops according to the business model of interest.

A node called a “touchpoint” is then allocated to each phase. Touchpoints represent both external contact events between customer and company or environment and the internal customer experiences they cause. There are two types of customer journey: those assumed while planning marketing activities and those resulting from plotting actual customer behavior histories. Practical CJMs provide perspectives for sharing better management of marketing activities throughout the organization. The current customer engagement process involving CJMs uses the planned journey as a default, transforming it into a journey based on actual measurements while measuring the progress, results, and outcomes of activities. Specifically, an unknown touchpoint observed in a direction deviating from an assumed transition from a known touchpoint to its intended subsequent touchpoint is called “inhibiting deviation,” and an unknown touchpoint observed in the direction of joining from the outside is called “activating deviation” (Halvorsrud et al., 2016). We can more precisely update journeys by incorporating these elements. We consider potential attitudes as underlying customer experiences that occur through a transition between touchpoints. Thus, the contribution of CJMs to marketing is an explainability that allows various decision-makers to perceive a more engineering-like approach to customer-oriented management. CJMs also contribute to providing a scope for innovative marketing automation. From the above, we suggest that CJM automation is suitable for problem formulation of human-AI decision-making collaboration in marketing.

### OTT Media Service

As a practical verification, this paper assumes the application to OTT media services (Farooq and Raju, 2019). Conventional entertainment content has been little more than a driver for the encouragement of distribution channels. There are various revenue structures, including on-demand, which charges for individual access, subscriptions, which allow unlimited viewing for a certain period of time, and advertising media models. In each case, the key to success is to continue attracting viewers to the provided content. The trend toward a business structure that combines online infrastructures with digital devices is not limited to entertainment content but is expanding to all marketing domains. Already, OTT media services are capturing behavior in customer activity logs practically and in real-time (Wayne, 2018), and data-driven decision-making systems are working to some extent for program organization, production, and promotion. Given such circumstances (Jenner, 2018), we consider OTT media services to be an appropriate experimental environment for the application of this research in marketing.

The domain-specific requirements for applying this research to OTT media services are as follows (Okazaki and Inoue, 2017). (1) Partial observations within short periods of time: In the case of a serialized drama, for example, an average of ten to thirteen episodes per season will be broadcast at regular intervals. One must learn to update the posterior forecast model sequentially according to the limited timing of each decision made from the viewing histories. (2) The potential and confounding of explanatory variables: Viewing attitudes can be fostered through complex interactions with public relations through such means as owned media, social media topics, and advertisements that are developed before broadcast according to stories for promotion and content. Therefore, latent explanatory variables must be identified while always assuming confounding covariates. (3) Appropriate granularity for data fusion: Customer contact histories for various marketing activities are often a data fusion of logs from different profit structures and strategies, making it is necessary to optimize to an observation granularity. (4) Process visualization: Because strategic decision-making involves a wide range of departments and the AI literacy of the decision-makers will vary, facilitation will be essential throughout the delivery period.

### Concept

**Figure 2:**
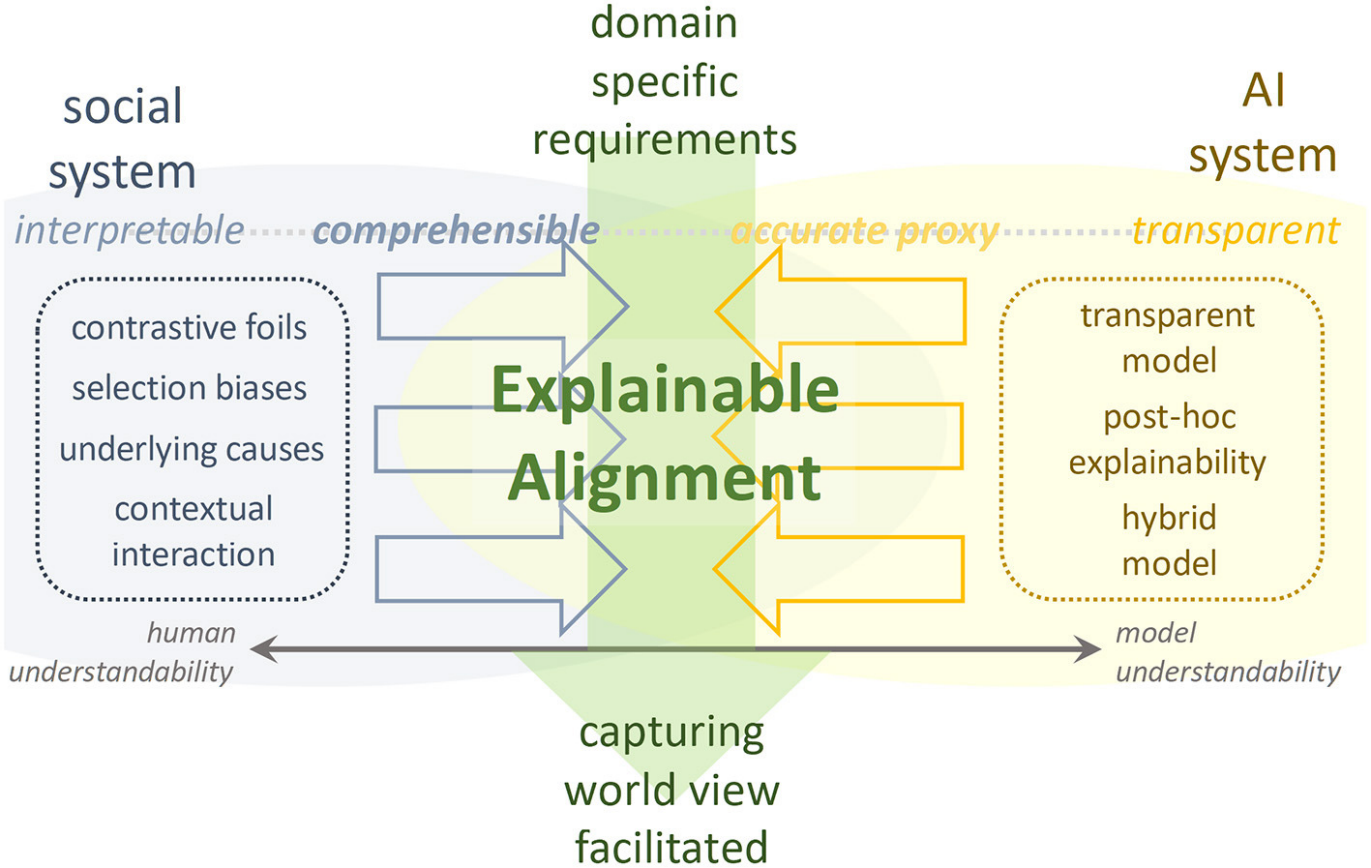
Explainable alignment. Overview of research idea.

We present explainable alignment as a concept for the AI system's collaborative facilitation with social systems. As Figure 2 shows, for an inverse problem that takes specific requirements to a domain as input and provides a facilitated worldview and acquired knowledge as output, the explainable alignment engages with the expertise in both systems while confirming their hypothetical perspectives. Social systems cast strategic perspectives for achieving the entire alignment as responsible questioning, and AI systems respond from a scientific perspective, thereby providing an autonomously correct answer. We continue to select and confirm viewpoints until this inverse problem is satisfied and the complementarity between the two are compatible, that is, until we can form an autonomous accountability for the solution in coordination with both.

An explicable alignment can be formulated as follows: *O*: CJM to be generated; I : data environment specific to OTT domain; *f*, *F*: decision-making frame from social system perspective; *m, M*: set of methods from AI system.

Explainable alignment is defined as finding the pair of (*f,m*) that best satisfies


$$\begin{array}{l} \left\{\left(\text{f,m}\right)|\text{f}\in \text{F,m}\in \text{M},\text{f}\left(\text{m}|I\right)\;=\;\text{O},\;\text{f}\;⊗\;\text{m}=\;0\right\} \end{array}$$

In other words, explainable alignment in this paper means solving the inverse problem of automating CJM under the requirements of the data environment of OTT media services, with AI methods working in a complementary way along the social decision frame. In this paper, we propose a combination of process mining on f^*^ and generative model and inductive logic programming on m^*^ as the optimal pair (*f,m*).

### Process Mining

Process mining (Van Der Aalst et al., 2011) is a general term for business process modeling and analysis that utilizes data mining by machine learning in these tasks. By extracting knowledge from event logs obtainable in the real world, we can discover, monitor, and improve the implemented processes. There are three directions for the analysis of process mining. The first is discovery, namely, the discovery of actual process models from event logs without the use of a priori knowledge. Next is conformance checking, which is the comparison of actual process models with the event logs they were obtained from and the matching of the model to reality, and vice versa. The third is enhancement, namely, the use of recorded information about actual processes to improve or extend the current process. In process mining, these three directions are simultaneously considered, requiring a process-centric analysis that can show concurrency. Even though the concept of process mining itself is in a framework for the social science field, the core of that concept belongs to the computer science field as data mining. In other words, although it is a tool for social systems, it is also an irreplaceable perspective for more explainable facilitation in that it opens doors for AI systems to observation, learning, knowledge, and reasoning.

### Generative Models and ILP

Generative models (Jebara, 2012) are a kind of modeling approach in machine learning. In general, this approach builds a hypothetical model from observation in advance and makes predictions and judgments by learning parameters and structures. Features behind the data are then extracted using computational methods such as inference and optimization. By clearly separating the solution process into inference and probabilistic modeling, uncertainty in the target system can be well expressed. In this way, the generative model that describes the generative process behind all observation data can create or increase unobserved data belonging to the class by sampling the probability density function. With better assumptions and purer observations, a model with more information can be obtained with fewer observations. On the viewpoint of process mining, a viewing log at OTT media services is a discrete event divided into periods that show release timings for content delivered at regular intervals. Therefore, the generative model discretely updates the probability model from a sequentially observed event log, and the vector of the probability density sampled at each time expresses the state.

One approach to extracting knowledge from this state transition is ILP (Muggleton, 1991), which is a machine learning approach that uses logic programming techniques defined at the intersection of inductive machine learning and logic programming. Focusing on the nature of inference rules, algorithm convergence, and the computational complexity of procedures, ILP extracts knowledge from experience through computational logic that constructs hypotheses from observations. Specifically, from a set of facts, expected results, and background knowledge, which are divided into positive examples to confirm and negative examples to infirm, an ILP system deduces whether a logical program is sound and complete. Because logic programming declaratively describes a problem, the logical formula that is finally derived can be shared, accumulated, and managed as explainable knowledge for various purposes. Furthermore, ILP can describe a state transition system as logical expressions, therein containing dynamics that dominate environmental changes.

Our approach thus uses ILP to extract knowledge from state transitions sampled by a generative model that learns features of interest based on event logs for viewers of the OTT media services. A graphical model of the hierarchical Bayesian approach, which provides the basis for the generative model, is computationally equivalent to a propositional logic formula with probabilistic measure given as an uncertainty, which has been pointed out as having weak descriptive power for general knowledge. This is because no general expression of knowledge itself is possible except by first-order predicate logic and its derivations. However, ILP successfully avoids this problem by using computational logic as a mechanism for expressing observations and hypotheses. Because the case at hand targets limited knowledge for viewing inherent programs in the specific domain of marketing, and furthermore because strategic decision-making presumes business literacy of the parties concerned, propositional logic can be adopted. However, if the discrete time argument is adopted in the propositional logic, the seamless utilization of the generative model for observations from the dynamic environment and ILP results in the state transition of the non-deterministic discrete event system, and knowledge extraction from it, as the first-order predicate logic. We thus have a probabilistic model that can be sampled from a small number of observations, a graphical model and prior distribution that explicitly incorporate the assumptions of causality and latency and can control hyperparameters to adjust granularity, a hierarchical Bayesian update to avoid overfitting and improve the accuracy for each observation, and a descriptive logic program that is inductively derived from the sampled state transitions. Based on the idea of reversing the task locality and combining only the effective parts of each technology, coordination between such a generative model and ILP can be assuredly prepared. By capturing the latency of variables from the constraints of the data structure of OTT media services (i.e., limited observations), we can create strategies that respond to the three process mining directions: discovery, conformance checking, and enhancement. In this way, process mining guarantees the facilitation required for the generative model and ILP in the field, providing process mining with tailor-made explainability for local issues. This is the complementarity that the explainable alignment arrives at for CJM automation from OTT media services event logs.

### Scheme

**Figure 3:**
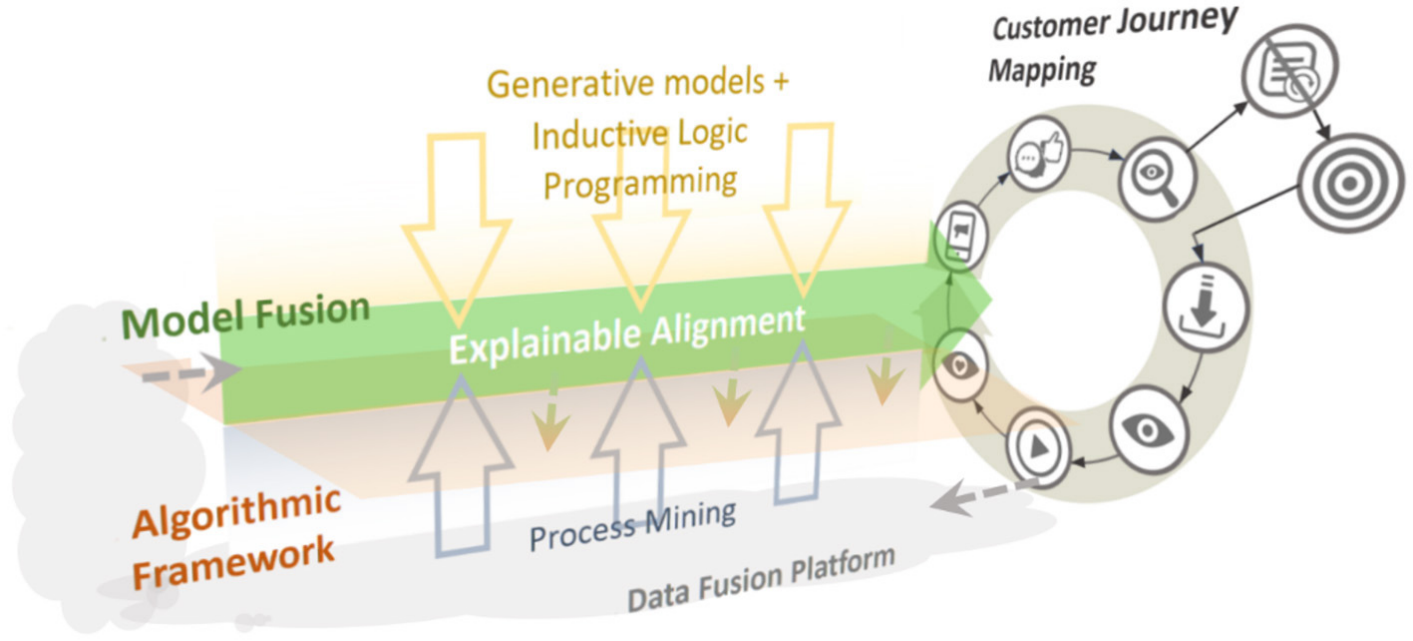
Methodological scheme of the proposed solution.

The scheme for a solution that automatically outputs CJMs along the explainable alignment is a loop structure in which four layers are combined as shown in Figure 3: an algorithmic framework, model fusion, CJM and rule extraction, and a data fusion platform.

A learning model and analysis technology are appropriately fitted to the algorithmic framework from the complementary viewpoints of the social system and an AI system derived in the alignment. This process is called “model fusion.” Through model fusion, a CJM and rules extraction is output from the observed data and fed back to the data fusion platform to improve the observation system. Then, taking as input the specific requirements on OTT media services, the complementarity achieved as an explainable alignment is a combination of process mining from the social sciences and knowledge extraction via generative models and ILP from the computer sciences.

## Method and Implementation

### Algorithmic Framework

The design concept of the framework is simultaneously for achieving a balance between the two types of black-box problems described above, knowledge extraction as a solution to inverse problems, and the design of explanations for the entire system.

**Figure 4:**
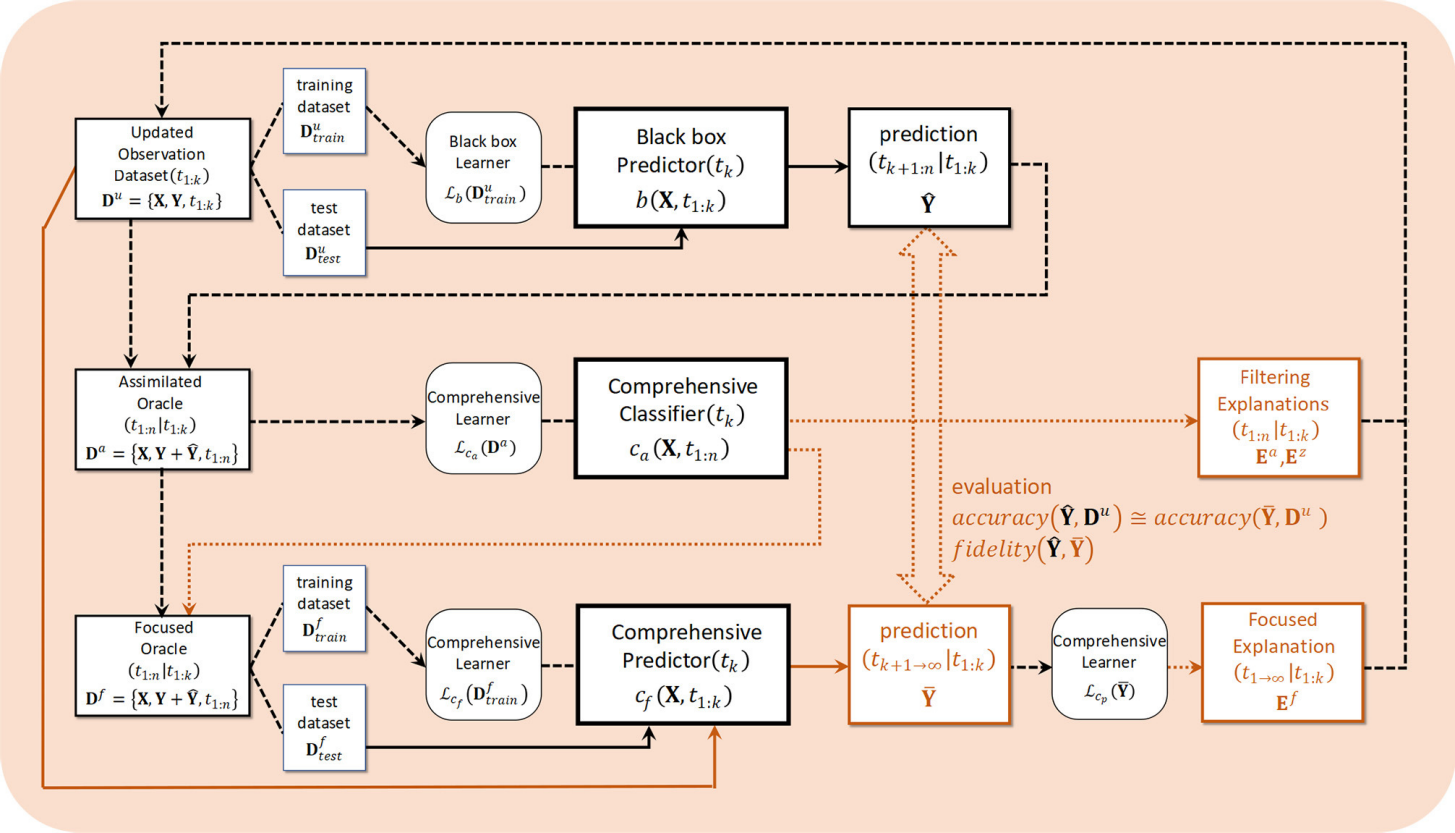
Algorithmic framework. The dashed arrows indicate processing, continuous lines indicates prediction and dotted arrows indicates explanation.

Figure 4 illustrates the algorithmic framework. At work in the upper row of that figure are the black-box learning $L_{b}$ and predictor *b*, which are capable of learning observation **D**^*u*^ obtained from the progress in content distribution and from sampling a subsequent prediction $\hat{\text{Y}}$. The input in the middle row is an assimilated dataset **D**^*a*^ that combines observation results with black box predictions. The generative model group $L_{c_{a}},C_{a}$ learns these and is responsible for extracting a feature quantity **E**^*a*^ for a latent variable. This corresponds to a solution for the inverse problem. The lower row in Figure 4 shows *post-hoc* explainability corresponding to the black box *b* in the upper row, and oracle data **D**^*f*^ resampled to include the target feature amount **E**^*a*^ to the extent possible among determined feature amounts are input. This predicts $\bar{\text{Y}}$ as CJM while visualizing the process through a comprehensible learner $L_{c_{f}}$ and predictor *C*_*f*_, then converting to knowledge **E**^*f*^ via the final learner $L_{c_{p}}$. This process corresponds to transparent box design (Guidotti et al., 2018).

### Discovery

Discovery in process mining is an important process that clarifies the object to be modeled under contrasting perspectives and selective biases in social systems, and in OTT media services there are few observations, latent variables, and confounding factors. To cope with these, generative model groups can reduce the required input dimensions by grasping probability densities in prior distributions and freedom in the hierarchical Bayesian graphical model. There are two potential features in modeling OTT media services. One is within-period viewing attitudes, which are units formed by viewers through experiences at various touchpoints before the distribution of subsequent episodes. The other is dynamic patterns in viewing attitudes, which are gradually formed within the flow of series distributions over multiple times. In our framework, the former is extracted by applying LDA, and the latter is extracted by applying a VAE.

LDA (Blei et al., 2003) is a representative generative model, called the “topic model.” Although the customer's behavior is reflected in the observation as a history, the object we want to act on is the latent attitude behind that behavior. LDA is a method for parsing latent semantics from sentences, and we can apply it to attitude extraction. The potential semantic relations called “words,” “sentences,” “document sets,” and “topics” in the topic model are replaced as-is by relations between individual unit behavior, their multi-period behavior history, and the set of viewers' history and latent attitudes in the modeling of OTT media services. It is also possible to represent how several different attitudes are potentially mixed behind a certain behavior, which is not possible with simple clustering methods. In this system, the dimension of marketing variables **D**^*a*^ is reduced in the dimension of latent attitudes through a learned LDA, and the viewing behavior of individual d for that week is output as a weight vector of filtered explanation **E**^*a*^ for each of the multiple different latent attitudes. An **E**^*a*^ converted into a weight vector for the attention latent attitude output by the LDA is converted into a grid image ${\text{e}}_{d}^{z}$, which is assigned a CMYK hue and saturation for color display. The resulting grid shows the individual's *n*th viewing attitude and can be regarded as a dynamic pattern for input to VAE as a 3 × 3 matrix image **E**^*z*^ constructed from the count and order of distributions. VAE (Kingma and Welling, 2013) is a three-layer neural network that uses the same data for the input and output layers, with a generative model that learns dimension-reduced features (the latent space) without supervision. The main advantage of VAEs is their explainable feature extraction during the learning process, rather than their use in classification. They generate a probabilistic distribution within this hidden layer as latent feature space, so unseen dynamic patterns can be sampled even from partial observation. Defining a pattern *l* of interest from the visualized features, we use an ${\text{E}}_{l}^{z}$sampled from within the latent space concerning that pattern and an observed **D**^*f*^ associated with that source as oracle data.

### Conformance Checking

Conformance checking in this system corresponds to the brown line connecting the upper and lower parts in the framework diagram in Figure 4. This is a verification of the explainability of the system itself as the XAI. Referring again to this figure, after determining the feature **E**^*a*^ that is the focus in the middle-row process, the data set **D**^*f*^ resampled from the original observation is simultaneously passed through the black box *b* in the upper row and the *post-hoc* explainability *C*_*f*_ in the lower row. While limiting to reflections of the focused features, evaluation and verification can be performed with two predictions $\hat{\text{Y}}$ and $\bar{\text{Y}}$, accuracy from comparison with the original observation **D**^*u*^, and *post-hoc* fidelity by comparing two predictions. Rather than trying to understand the inner states using a model-agnostic method, our goal is to suggest a decision-making policy from the estimated model itself. We do this by selecting a pair of black box and *post-hoc* explainability as model-understandable methods in which the structure of the models is suited to the social decision-making process. Our hybrid transparent black-box method thus uses a LSTM as the black box and the HMM, which is a typical discrete state space model, for *post-hoc* explainability.

LSTM (Murdoch and Szlam, 2017) is a typical RNN that currently is most commonly applied when processing continuous data, such as time series predictions. In our system, LSTMs cumulatively and incrementally input the unit viewing behavior **X**_*n*_ observed in the *n* th week of delivery to generate predictions for the remaining *Nn* occurrences. A dataset in which the input values until the *n*th episode **X**_1:*n*_ and the remaining predicted values **X**_*n*+1:*N*_ are assimilated is considered as the oracle. HMM (Baum and Petrie, 1966) is a finite-state machine having a probabilistic model and is widely used in modeling for time-series data from a wide variety of fields. Content viewing behavior in OTT media services often show different tendencies between the first and second halves of occurrence time series. Moreover, attitudes behind touchpoints and content viewing cannot themselves be directly observed. As a characteristic of serial dramatic productions, previous viewing drives current viewing, and we can assume that there is a dependency between adjacent latent variables and that they are conditionally independent. In that sense, an HMM is a remarkable method in that for the first time it allows for the modeling of CJM solely from observations as complicated realities of human psychology toward such purchases.

### Enhancement

Our alignment converts the CJM that is finally output into knowledge and continues updating it. The readability of symbolic expressions that enables inference for various uses enhances the CJM. In models dealing with time series, such as LSTM cell-state, context information, and HMM latent series **s**_*t*_, the latest knowledge always exists within the state transition. To extract this knowledge, we use an ILP field technique called LFIT.

LFIT (Inoue et al., 2014) is unsupervised learning that automatically learns system dynamics from observation data obtained from a certain dynamic environment. Normal logic programming (NLP) is a set of rules in the form

(1) $$\begin{array}{r} A\;\leftarrow \;A_{1}\;\wedge \;\ldots \;\wedge \;A_{m}\;\wedge \;\neg \;A_{m\;+\;1}\;\wedge \;\ldots \;\wedge \;\neg A_{n} \end{array}$$

where *A* and *A*_*i*_ are atoms and (1 ≤ *i* ≤ *n, n* ≥ *m* ≥ 0). In a given rule *R* in the form of (Equation 1), *A* is the head of *R*, represented by *h*(*R*), and the conjunction on the right of the ← is the body of *R*. The set of literals appearing in the body of *R* is represented by *b*(*R*) = {*A*_1_, … *A*_*m*_, ¬*A*_*m*+1_, …¬*A*_*n*_}, an atom appearing as a positive literal in the body of *R* is $b^{+}\left(R\right)=\left\{A_{1},\ldots ,A_{m}\right\}$, and a negative literal is expressed as $b^{-}\left(R\right)=\left\{A_{m+1},\ldots ,n\right\}$. All atoms appearing in NLP are called a “Herbrand base B.” Herbrand Interpretation *I* is a subset of B and is an assignment of truth values to each atom. Given a rule *R* and an interpretation *I*, if *b*^+^(*R*) ⊆ *I* and *b*^−^(*R*) ∩ *I* = ∅ imply *h*(*R*) ∈ *I*, *I* is said to satisfy *R*. A consistent Interpretation *I* is a model of *P* where *I* satisfies all rules in *P*. When considering a logic program *P* and interpretation *I*, the *T*_*p*_ operator defined as

(2) $$\begin{array}{r} T_{p}\left(I\right)\;=\;\left\{h\left(R\right)|R\in P,b^{+}\left(R\right)\subseteq I\;and\;b^{-}\left(R\right)\cap I\;=\;\emptyset \right\} \end{array}$$

is a mapping *T*_*p*_: $2^{B}\to 2^{B}$. Time is used as an argument for expressing the dynamics of a system that changes over time. That is, the atom *A* at time t is regarded as *A*(*t*), allowing us to rewrite the rule of Equation (1) as the dynamic rule

(3) $$\begin{array}{r} A\left(t\;+\;1\right)\;\leftarrow \;A_{1}\left(t\right)\wedge \ldots \wedge A_{m}\left(t\right)\wedge \neg A_{m\;+\;1}\left(t\right)\wedge \ldots \\ \;\wedge \neg A_{n}\left(t\right) \end{array}$$

This means that when *A*_1_, *A*_2_, …, *A*_*m*_ are all true at time *t*, and *A*_*m*+1_, *A*_*m*+2_, …, *A*_*n*_ are all false at *t*, then *A* is true at *t* + 1. By writing this in the form of Equation (3), the *T*_*p*_ operator can simulate state transitions of the dynamic system. We focus on state transitions between two adjacent time points. When the interpretation *I* reflects the system state at time *t*, the state *J* of the system at time *t* + 1 is another interpretation. In that case, the state transition from *I* to *J* in the dynamic system can be expressed as (*I, J*). Given a set of interpretation transitions *E*, an algorithm that learns logical program *P* satisfying *T*_*p*_(*I*) = *J* for all the transitions (*I, J*) ∈ *E* is called LFIT. The premise for LFIT to arrive at the correct logical conclusions is that Herbrand interpretation *I* is fully observed. In other words, the soundness and completeness of the rules that appear can only be presumed if transitional relationships for all of the many possible worlds are included in the observations. By contrast, like other observations in the real world, observations of OTT media service viewing histories are always uncertain and incomplete. State transitions obtained from HMM are thus non-deterministic and LFIT cannot be directly applied. We therefore introduce a BN as a concept for dynamic systems that connect actual state transitions and logical programs.

**Figure 5:**
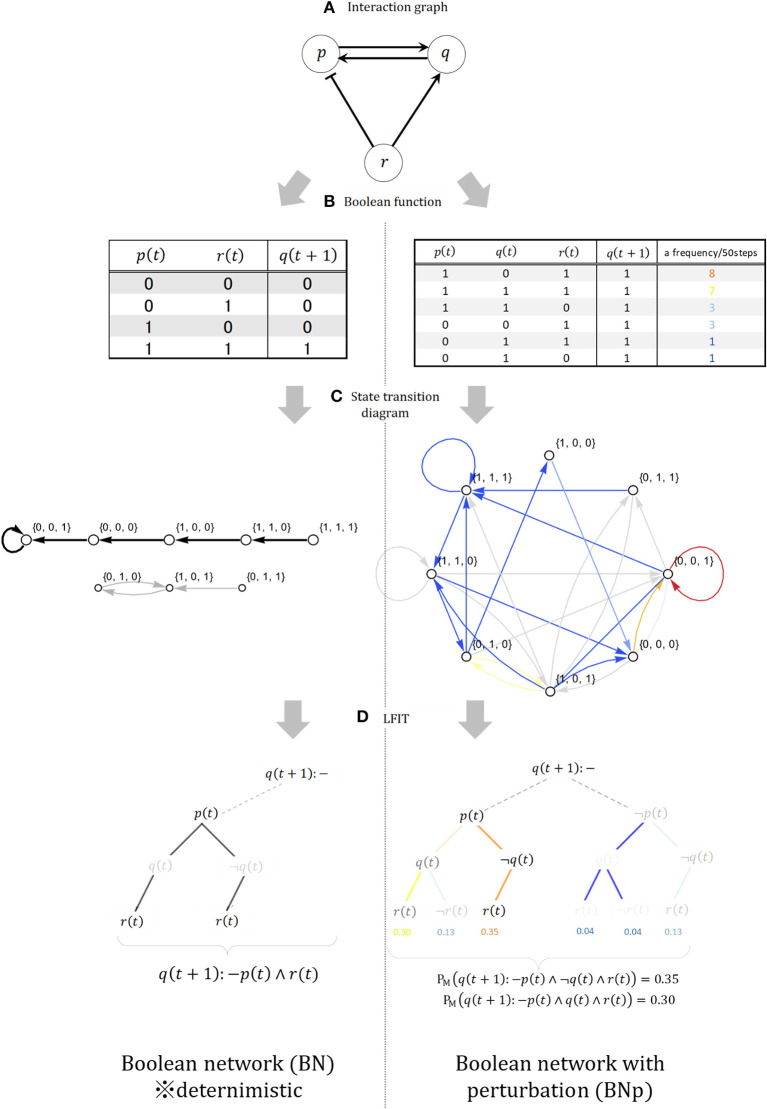
**(A)** Interaction graph. **(B)** Deterministic and non-deterministic Boolean function. **(C)** State transition diagram. **(D)** Example of interpretation transition (LFIT) output.

A BN (Kauffman, 1969) *G*(*V, F*) is a graph representation consisting of a Boolean function set *F* = {*f*_1_, …, *f*_*n*_} associated with the set *V* = {*x*_1_, …, *x*_*n*_} of finite binary variables (nodes). *x*_*i*_(*t*) ∈ {0, 1} (*i* = 1, …, *n*) represents the value of node *x*_*i*_ at timestep *t*, and the network state is the vector **x**_*t*_ = {*x*_1_(*t*), …, *x*_*n*_(*t*)}. If there are 2^*k*^ possible and clear states at each timestep, and *x*_*i*_1__, …, *x*_*i*_*k*__ are a group of nodes that directly affect *n*_*i*_, called “*x*_*i*_ regulation nodes,” then in the next timestep *t* + 1 the state of node *x*_*i*_ is *x*_*i*_(*t*+) = *f*_*i*_(*x*_*i*_1__(*t*), …, *x*_*i*_*k*__(*t*)). The BN is drawn by an interaction graph like that shown in Figure 5A, but its strict regulatory relations are expressed only by a Boolean function like those shown in the upper-left of the figure. From any BN, we can calculate a state transition diagram showing synchronous transitions of all nodes between *x*_*i*_(*t*) and *x*_*i*_(*t* + 1), as shown at the middle-left of Figure 5. The LFIT calculation process includes the BN comprising variables {*p, q, r*}, the truth table of the Boolean function for node *q*, the state transition diagram, and a combinatorial tree for logic programming. The Boolean functions *f*_*p*_, *f*_*q*_, *f*_*r*_ for each node *p, q, r* of BN are *f*_*p*_ = *q*, *f*_*q*_ = *p* ∧ *r*, and *f*_*r*_ = ¬*p*. For example, node *p* activates node *q* while simultaneously suppressing node *r*. The BN is thus deterministic, the only potential uncertainty being the choice of the initial state of the network. Once given an initial state, the BN will transition within a finite number of steps to either a fixed state or a set of states that iteratively circulates forever. As per Inoue (2011), the BN *G*(*V, F*) can convert transition τ(*G*) to the propositional logic NLP π(*G*) by removing the time argument from each literal *A*_*t*_ appearing in τ(*G*). Thus, in the *T*_π_(*G*) operator, the trajectory of *G* can be simulated from any state. This allows the application of LFIT to the state transitions of finite automata as long as the model is deterministic.

Next, we launch the BNp (Lähdesmäki et al., 2006) for application of LFIT to non-deterministic state transitions based on uncertainties and incomplete observations. The BNp is a BN that introduces probability. Any transition can cause the network to move away from its current trajectory to a randomly chosen state, from which it can start following the orbit as an updated initial state. The perturbation mechanism is modeled by introducing the parameter π (0 < π < 1) and the perturbation vector γ = (γ_1_, …, γ_*n*_), where γ_1_, …, γ_*n*_ are binary random variables with independent and identical distribution, and Pr(γ_*i*_ = 1) = π and Pr(γ_*i*_ = 0) = 1 − π for all *i* = 1, …, *n*. The choice of state transition depends on the realization of the current perturbation vector given at each transition step. If the state of the network at time *t* is *x*(*t*) ∈ {0, 1}^*n*^, then the next state *x*(*t* + 1) is *f*(*x*(*t*)) or *x*(*t*) ⊕ γ(*t*). Note that ⊕ is component-wise modulo 2 addition, and γ(*t*) ∈ {0.1} is the realization of the perturbation vector for the current transition. *x*(*t*) ⊕ γ(*t*) means that *x*_*i*_ (with *i* = 1, …, *n*) changes its value if γ_*i*_ = 1 and does not change if γ_*i*_ = 0. The right side of Figure 5 shows each property of the BNp; for example, the truth table at the upper-right is generated by adding the perturbation parameter π = 0.25 to the deterministic BN and shows the types and numbers of transitions actually occurring with node *q* as the head. Taking the example of starting from (1, 1, 1) in a BNp and advancing 50 steps, the colors indicate the frequency of state transitions. Compared with that for the BN, we can see that state transitions in the BNp are randomly added to the frequent transitions in the BN as various deviations. As shown in the combinatorial tree at the lower right of Figure 5, learning from state transitions in a BNp requires considering weights on the bodies that are added in various ways according to the generation of nondeterministic states. However, one feature of a BNp is that such random perturbations allow the network to escape the attractor, so these perturbations make the system reachable in any current state from a single transition. As a result, the dynamics of a BNp are equivalent to an ergodic first-order Markov chain (Tierney, 1996), such that the BNp has a unique stationary and steady-state distribution with nonzero probabilities assigned to each state. In subsequent state transition pairs (*I, J*) where *I* = *J*, according to Fenstad's theorem (Fenstad, 1967), the density distribution of the joint probability *Pr*(*I, J*) consisting of the product of the steady-state distribution and the transition probability matrix is a probability measure for a logical program that can be induced from (*I, J*). The NLP with a probability measure obtained from this transition pair is a group of competing explanations for the BNp as a stochastic event in distribution semantics (Sterling, 1995), using the semantically unaffected LFIT algorithm GULA (Folschette and Ribeiro, 2020). Among them, the explanation with the highest probability measure is the best explanation. Importantly, because the HMM is an irreducible first-order Markov chain, as with a BNp, an NLP with a probability measure can be obtained from the state transition after advancing the timestep and reaching the steady-state. Thus, with the launch of the BNp and the general-purpose LFIT algorithm, we can extract knowledge even from the non-deterministic state transitions of an HMM. The models combined in terms of both theoretical and practical implication are summarized in Table 1 below in the order of their use in processing. As Table 1 shows, our proposed method matches a management technique called process mining with a machine learning method called ILP from generative models. For the various literacies of stakeholders, process mining shows the current coordinates of processes leading to decision-making, thus supplying suggestions to the machine learning side, and the generative model and ILP are used to perform computational processing that advances the coordinates and outputs consequential and necessary knowledge in a visible form.

**Table 1:** Summary table for managerial and theoretical implication.

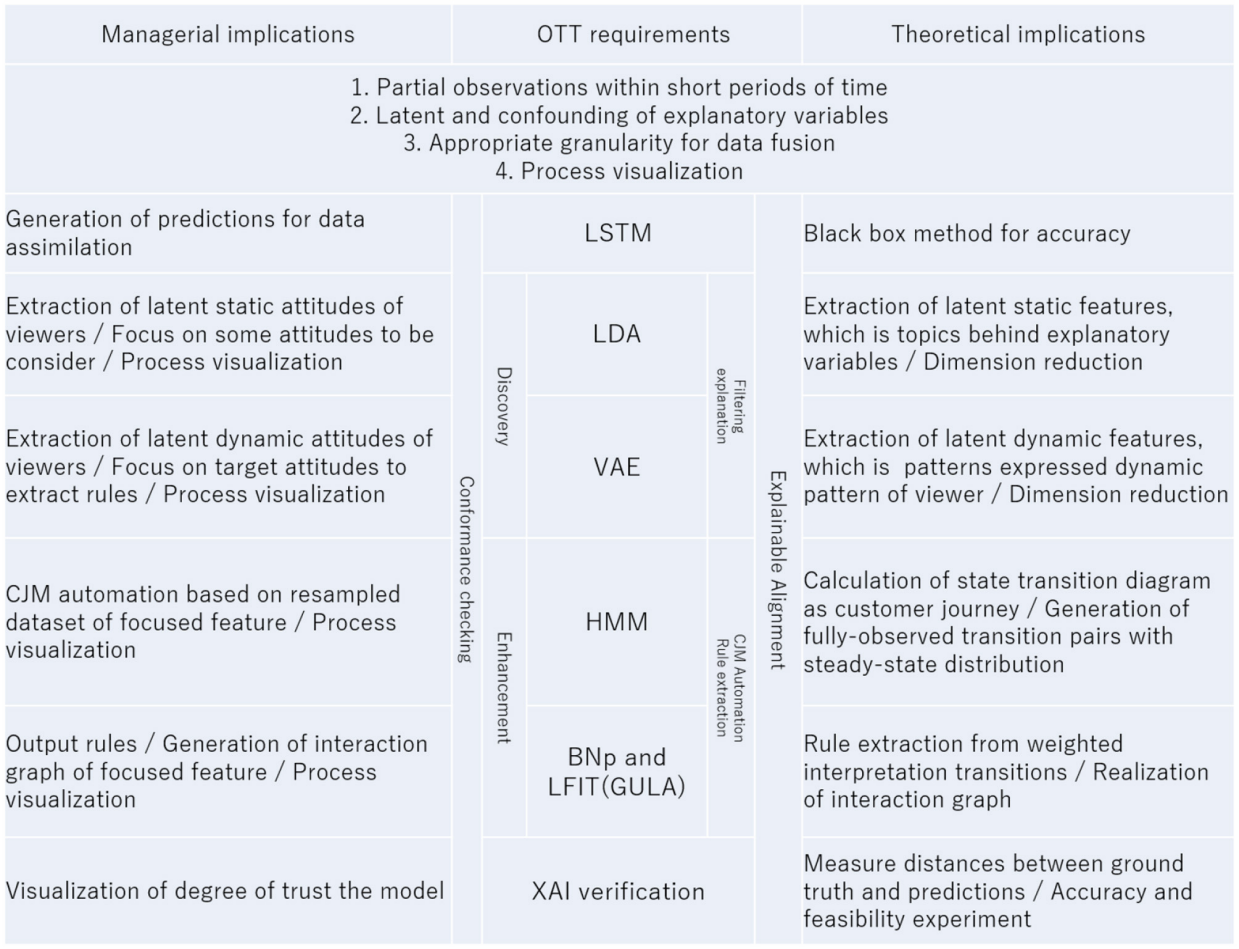

## Experiment and Results

### Data Environment and Preprocessing

Arrieta et al. (2020) categorizes data fusion into three types according to processing. Namely, there is a data-level fusion that combines raw data from different sources, a model-level fusion that combines each model learned from a subset of the combined data, and a knowledge-level fusion that merges, using ontologies or other expression formats, to complement or improve the originally provided knowledge set. The platform, as an observation environment, is the first data-level fusion. We use a previously implemented OTT media service as this observation environment. The environment is a fusion format called the “centralization approach,” in which data locally acquired at each observation node are sent to a centralized processing system and are merged together.

Specifically, the touchpoints for content viewing via OTT media services and marketing activities via the Internet are acquired from a single source in units of minutes, and we utilize a data environment called “esXMP” (Video Research Inc, 2021) that integrates seven data types from smartphone terminals, seven data types from television receivers, and four profile-related data types into a master database. The structure of the observation data prepared for this study comprised four dummy variables, **x**_1…4_, related to the dynamic viewing attitude **x**_*d,t*_ of individual d at time t, and nine explanatory variables, **x**_5…13_, related to the touch point, which together form a thirteen-dimensional binary vector. For discretization, the week from the end of distribution of some targeted content to the end of distribution of the next content is regarded as a single period. Following the viewing criteria used by the actual business, each variable has a dummy variable that records whether the content was viewed in the previous week and whether it was viewed this week, along with whether the viewer engaged with marketing activities before the content distribution within that week.

We used the behavioral histories of touchpoints related to a 60-minute serial drama rebroadcast during the prime-time 22:00 slot. This drama series, a popular romantic comedy, first aired in 2016. It became a social phenomenon, resulting in the creation of various types of user-generated content, such as buzzwords and dances. The drama targeted a broad range of viewers, aged 15–50 years, and it was easy to access related marketing from personal smart devices. Six broadcasts had already been completed, while the first few episodes of the next new program had been completed and were waiting to be broadcast. The broadcasters were faced with questions such as when to switch to the new program, whether doing so would betray fans of the current drama, and what measures must immediately precede the switchover.

For training, we used updated observation vectors **D**^*u*^ ∈ {0, 1}^13^ for 1,058 persons from esXMP, accumulated from the first to the sixth broadcast, and an LSTM with 50 layers over 120 epochs learned them. Each window was trained to predict a fourth week by incrementally moving the history for three consecutive weeks. We adopted a known approach for handling discretized training examples. We used an LSTM that had already been trained in this way as the black-box learning $L_{b}$ and predictor *b*. We input another 353 viewers **D**^*u*^ as test data, and fed back output predictions to produce $\hat{\text{Y}}$, forecasts of weekly touchpoints and viewing behaviors of the main story for the seventh to the ninth episodes. The original **D**^*u*^ and $\hat{\text{Y}}$ are assimilated to produce the oracle data **D**^*a*^ for 353 viewers for weeks 1–9. This is the source of the latent features and the input for post-hoc explainability in XAI.

### Filtering Explanations

**D**^*a*^ is effectively a tensor of order 3 with dimensions for viewers ID 1–353 with behavioral histories {0, 1}^13^ over viewing weeks 1–6, but since we know that the history over a viewing week will be sparse, we convert the behavioral history to binary in advance to increase computational efficiency. This allows for conversion of the entire viewing history to a matrix, with each individual week converted to a vector of binary instances. By considering this vector as a sentence comprising six words, we can apply the methods of latent semantic analysis from the field of natural language processing. The assimilated oracle **D**^*a*^ is input into LDA and is trained. In the trained LDA, a topic is equivalent to a latent attitude consisting of a combination of touchpoint variables, and the topic (i.e., latent attitude) is inductively and qualitatively interpreted and annotated while comparing the probability of occurrence of a group of touchpoint variables specific to an attitude (Table 2) from the topic distribution. Since the probability gives the likelihood of latent attitudes across the entire sample, we select up to four latent attitudes that should be prioritized.

**Table 2:** Definition of variables.

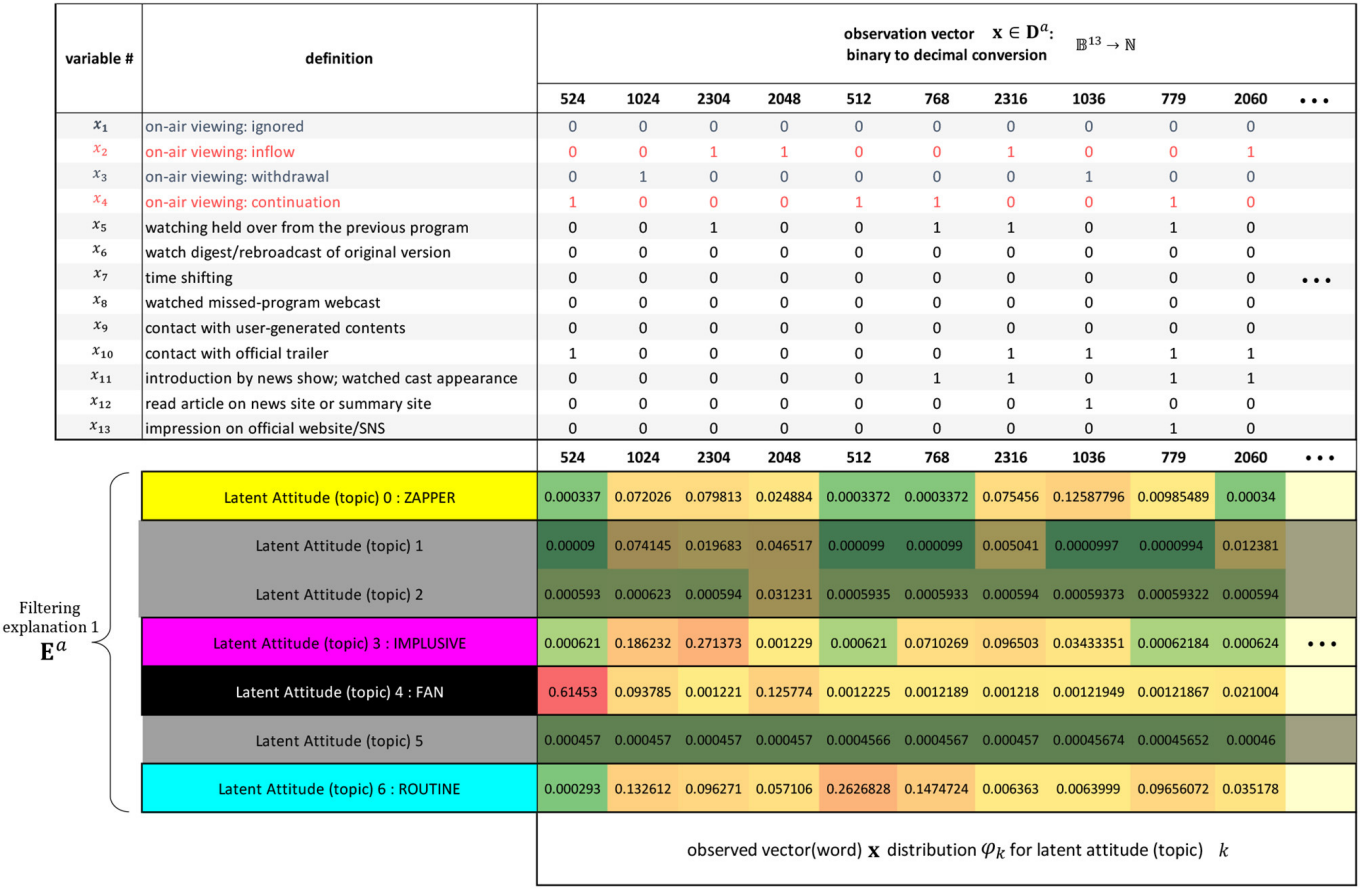

*The color of latent Dirichlet allocation (LDA) topics indicates their corresponding CMYK color assignment. Owing to limited space, only the 10 most important pattern numbers are included in the chart*.

**Figure 6:**
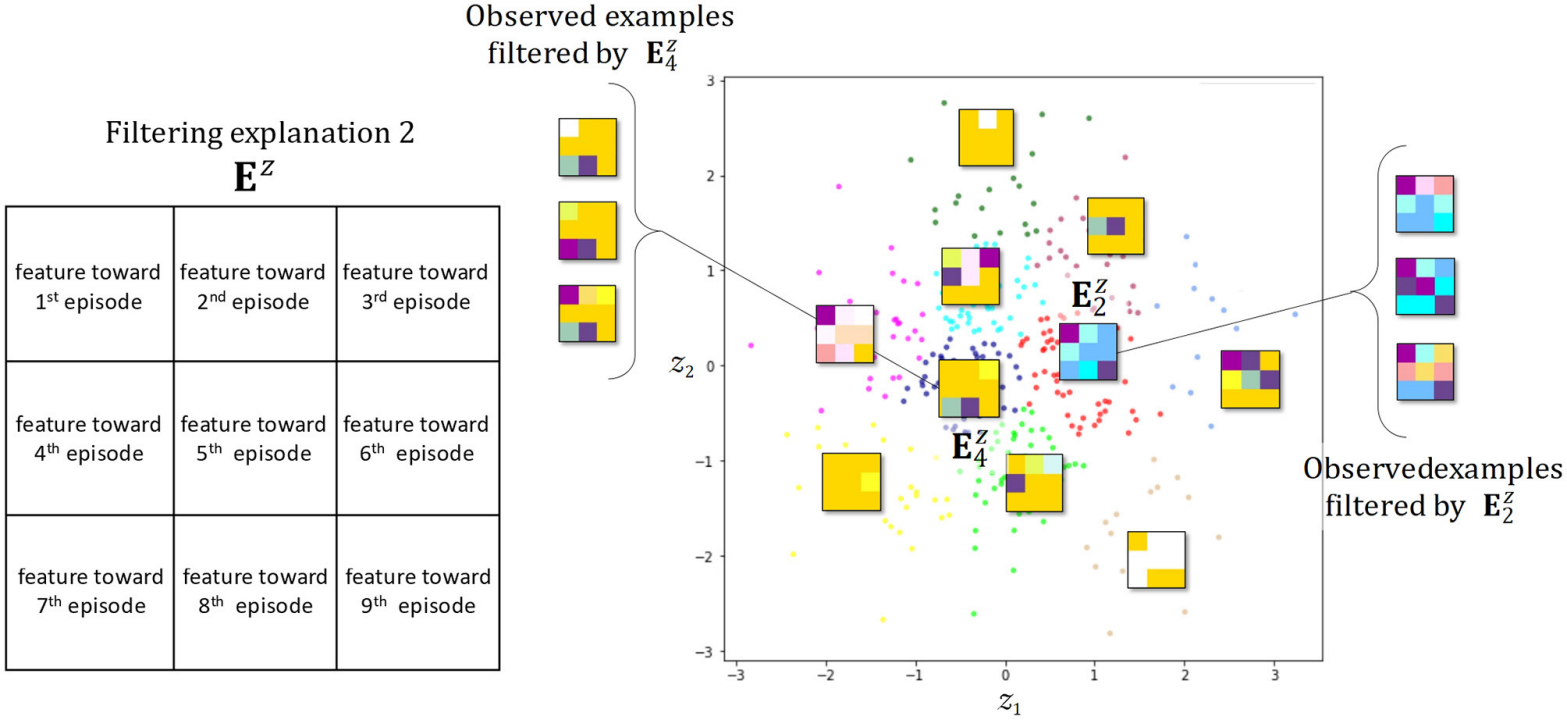
Filtering explanation by visualizing the variational autoencoder (VAE) latent space and manifold. Representative color grids of each cluster are shown.

This process is the stage at which the explanatory potential of static latent attitudes among latent features can be examined. To specify the number of topics, we select and use the model for it that possesses minimum perplexity and maximum coherence from a set of 2 to 50 trained models, designated in advance. Transforming the oracle **D**^*a*^ into a real-number vector representing up to four selected latent attitudes yields the explanatory vector **E**^*a*^, with $⋃{\text{e}}_{i,1:9}^{a}\in {\left(0,1\right)}^{4}$. **E**^*a*^ contains the complex dynamics of latent attitudes from the first time to the final prediction. In other words, this process is the stage at which the explanatory potential of dynamic latent attitudes can be examined. The personal history ${\text{e}}_{d}^{a}$ for individual *d* over nine viewings comprising the explanatory vector **E**^*a*^ is transformed into a 3 × 3 matrix of 4-dimensional vectors with latent attitudes stored in each cell, and this is input into the next feature extractor, the VAE. The explanatory matrix **E**^*z*^ is the 3 × 3 feature matrix data that are generated with a Gaussian distribution, which is the latent space of the learned VAE in Figure 6. If real values for the four prespecified latent attitudes are used as arguments for CMYK or some other color function, the dynamic viewing pattern becomes a 3 × 3 color grid, with features clearly shown. This allows an intuitive consideration of noteworthy viewing trends and tendencies to contact touchpoints across the entire broadcast distribution period.

### CJM Automation

Using the explanatory matrix **E**^*z*^ as a filter, only viewing patterns considered to be similar and predicted viewing histories are re-collected to obtain the oracle set **D**^*f*^ that is our focus. We evaluate the HMM from this **D**^*f*^. The number of latent states is predicted in advance in the range of 2–20, and the posterior Bayesian information criterion (BIC) is used to narrow down to the model with the highest accuracy. The resulting state transition diagram representation is the target of our system, namely, realizing a CJM that generates the viewing pattern. Annotating the emission probabilities of viewing pattern vectors **D**^*a*^ ∈ {0, 1}^13^ facilitates interpretation of the latent states. This process is CJM automation. Rather than mindlessly drawing arrows to a target state, we come to consider where invisible cliffs rise up in the minds of customers or viewers.

### Rule Extraction

When extracting logical rules from CJMs that generate viewing patterns of interest, we take advantage of the fact that the state transition diagram of the HMM obtained by observations from nature is an ergodic first-order Markov chain, and that recursive sampling always leads to a steady-state distribution. Once a steady-state is reached, the transition function from the subsequent state *I* to the next state *J* is the same as the *T*_*p*_ operator in a BNp. In this case, the discrete joint probability that is a product of the stationary distribution *p*(**S**) and the transition probability matrix is the probability measure of the state pairs of the steady-state. The LFIT from state pairs between steady-state differs from that between deterministic operation in that the number of detected state pairs is proportional to a perturbation parameter γ, and a conflicted relation between the rules increases accordingly.

In our problem setting, we are considering discrete probabilistic systems. Probabilistic mechanisms introduce non-determinism and different ratios of occurrence for each possible state transitions. GULA only learns which next values a variable may take in a given configuration, but not their probabilities. For example, by giving the transition ({*a*^1^, *b*^0^, *c*^2^}, {*a*^0^, *b*^2^, *c*^1^}) to GULA, we are requesting it to learn the claim: “*a*^0^ is possible after {*a*^1^, *b*^0^, *c*^2^}.” But to represent a probabilistic dynamical system, we expect rules that state: “There is *X*% chance to have *a*^0^ after {*a*^1^, *b*^0^, *c*2}.” Thus, in order to use GULA to learn probabilistic rules, we can encode the probability in the transition, for instance: ({*a*^1^, *b*^0^, *c*^2^}, {*a*^0^, 75%, *b*^2^, 100%, *c*^1^, 100%}). Technically, *a*^0^, 75% and *b*^2^, 100% are just regular symbolic atoms with a different domain. Given such transitions, GULA would learn rules like *a* 0.75%←*a*^1^, *b*^0^, *c*^2^ meaning “When *a* has value 1, *b* has value 0 and *c* has value 2 in the current state, *a* takes value 0 in the next state in 75% of the cases. Therefore, the GULA algorithm counts occurrences of all pairs and uses their frequencies as a probability measure to determine which rule to prioritize. The resulting BN, as an interaction graph, is a diagram that shows causal relations and those activating or inhibiting dynamic relations among the, at most, 13 nodes that are the explanatory variables. Interaction graphs facilitate understanding of the whole picture, which can be difficult to do in terms of rules expressed as individual logic programs.

## Results

As shown in Table 2 again, a result of LDA learning with assimilated oracle data **D**^*a*^ and a range of topic counts from 2 to 50, since the best pair was between lower perplexity and higher coherency the decided number of topics as hyperparameters was 7. The lower portion of Table 2 shows whether there is a high probability that each latent attitude (topic) will appear in the touchpoint contact pattern (a 13-dimensional vector converted to binary) in this model. While some patterns, such as #2048, are markers for all latent attitudes, others, such as #524, appear only in specific attitudes. From here, we annotate each latent attitude. For example, we can refer to an attitude that has a high probability of #2316, leading to inflow viewing associated with contact with a trailer, as the “zapper” or “impulsive” type, or attitudes showing high values for #768, leading to viewing previous programs, as the “routine” type. In the present study, we selected four interesting attitudes: #0 and #3, leading to disengagement, and #4 and #6, leading to promotion.

Figure 6 shows again the dynamic features superimposed as a 3 × 3 color grid distributed on a 2-dimensional manifold representation of the latent space Z for a VAE that has learned the explanation vector **E**^*a*^ from a selected combination of latent attitudes annotated {“routine”, “impulsive”, “zapper”, “fan”}. The number of dimensions of the latent space is a hyperparameter of the VAE, but we chose a two-dimensional manifold representation to account for the explainability of the feature. A CMYK color function is assigned to each combination of latent attitudes so that the first three views are arranged in the top row, the fourth through sixth in the middle row, and the seventh through ninth in the bottom row. With this color grid, we can see how the four static viewing attitudes change in a complex manner as the distribution progresses. For example, ${\text{E}}_{4}^{z}$ in Figure 6 shows how viewership increases toward the end as a result of promotion programming, despite contention between the numbers of departing and joining viewers. From the latent space of VAE, for example, we can see by generating samples that there can be an unknown observation pattern which is more easily found around the already detected ${\text{E}}_{2}^{z}$ and ${\text{E}}_{4}^{z}$. By layering hierarchical clustering and the like on top of this manifold representation, we can freely induce the number of features and express how features are related to each other to create new patterns.

Subsequently, we evaluated an HMM to elucidate the dynamic viewing patterns of interest. The number of latent states as hyperparameters was previously estimated to be in the range 2–20, and the model with the five latent states with the lowest BIC value was adopted. Table 3 shows the emission probability of the touchpoint contact patterns (binary vector) that feature each of the latent states 1–5. As in the case of LDA, we annotate each state as a representative or inductive contact pattern. For example, both *T*_1_ and *T*_2_ are classified into #4108 as a negative reaction, but one has the property of #2304 and the other of #2316, so we can give *T*_2_ a name like “passive attention.”

**Table 3:** Focused oracle and the annotation of the hidden Markov model (HMM) (touchpoints).

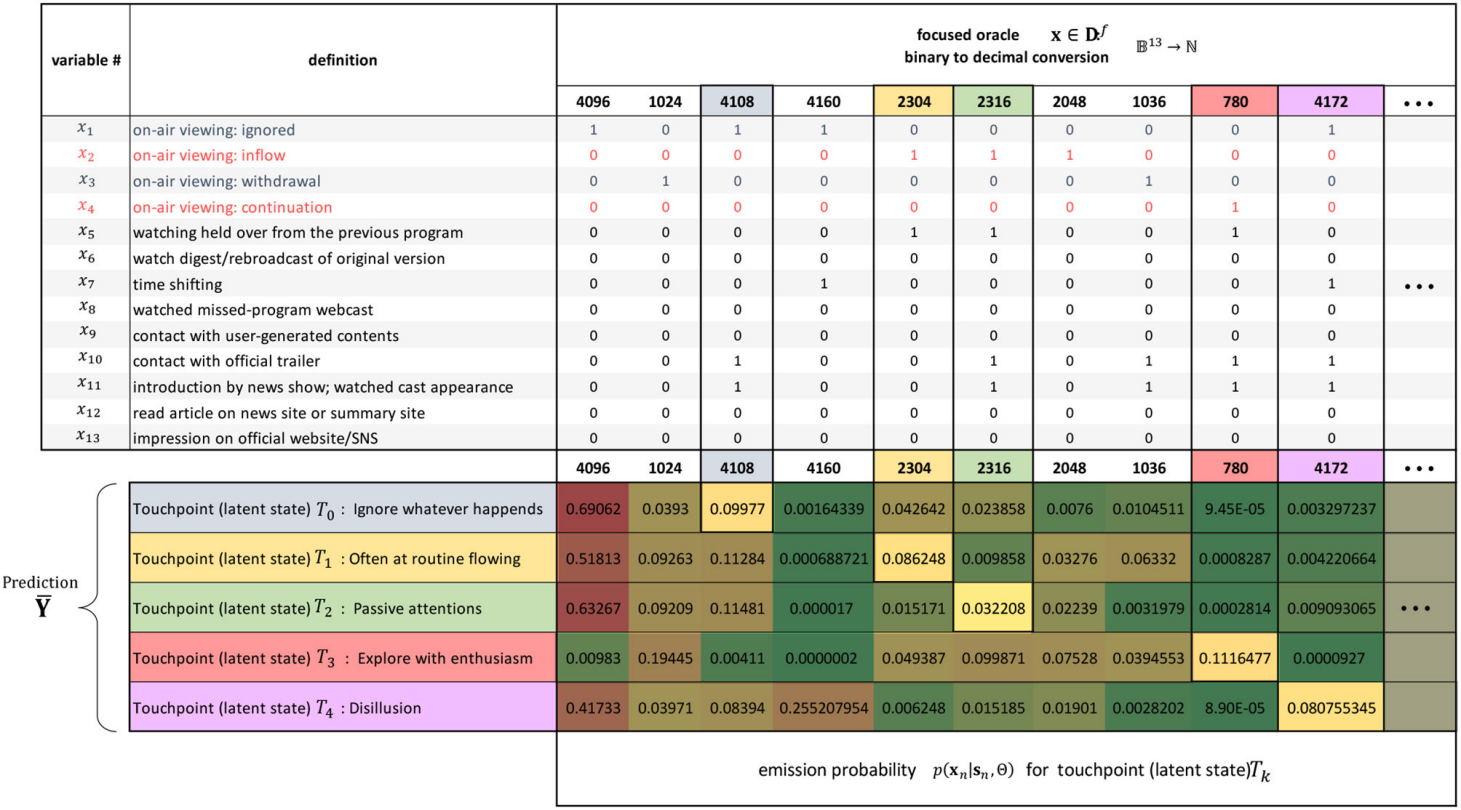

*The bottom matrix is the emission and the selected pattern number is emphasized with highlight color. Owing to limited space, only the 10 most important pattern numbers are included in the chart*.

**Figure 7:**
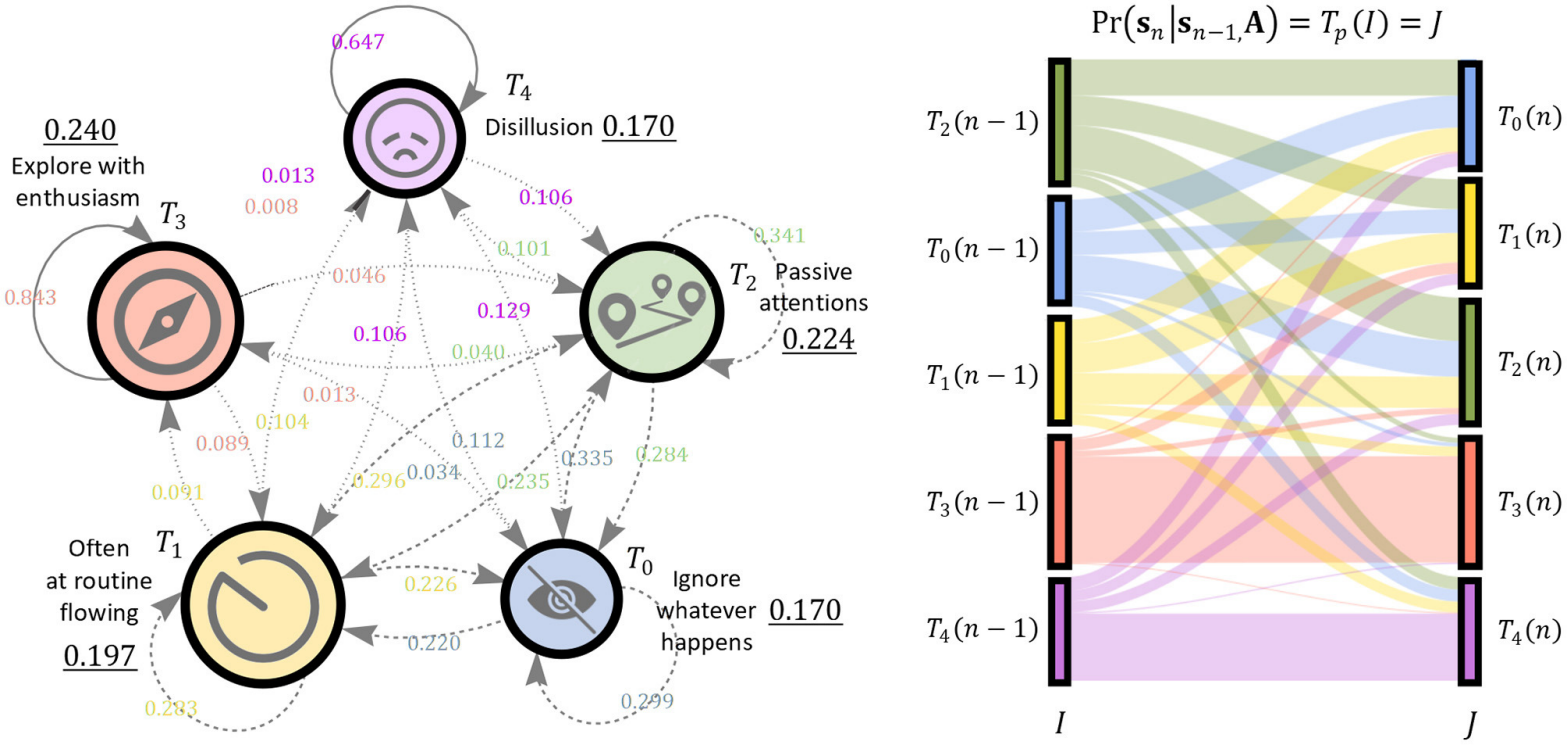
Left: Result of Customer Journey Mapping. Initial probability is illustrated by numbers with underline and other numbers are transition probability. Right: Sankey Diagram. Transition between steady states is shown, note that the state distribution is similar on both side of transition.

The latent states interpreted in this way are associated with a fixed transition probability. As shown in Figure 7, another property is that content viewing behavior strongly expresses the effects of feedback and self-loops, and it is advantageous to be able to predict areas that cannot be analyzed by directed non-cyclic graphs such as Bayesian networks. The appended Sankey diagram on the right in Figure 7 intuitively shows how the joint probability between transition pairs following the stationary distribution integrates to 1, and also that this value corresponds to a probability measure in possible world semantics. GULA learns these inter-transition pairs as interpreted transitions.

Table 4 shows the rules extracted by LFIT (GULA) from transition pairs of stationary distributions with the above-described probability measures. The more likely the NLP is, the closer we get to a rule that would have been true if determinism had originally been assumed in the model. By selecting the best on the list as a candidate for each *h*(*R*) with the explanatory variables appearing in the observation, and realizing an interaction graph from it, we can see that this model is suitable for a first-order Markov chain of deterministic uncertainty with perturbations. In other words, a combination of a BNp and HMM.

**Table 4:** Extracted rule of GULA.

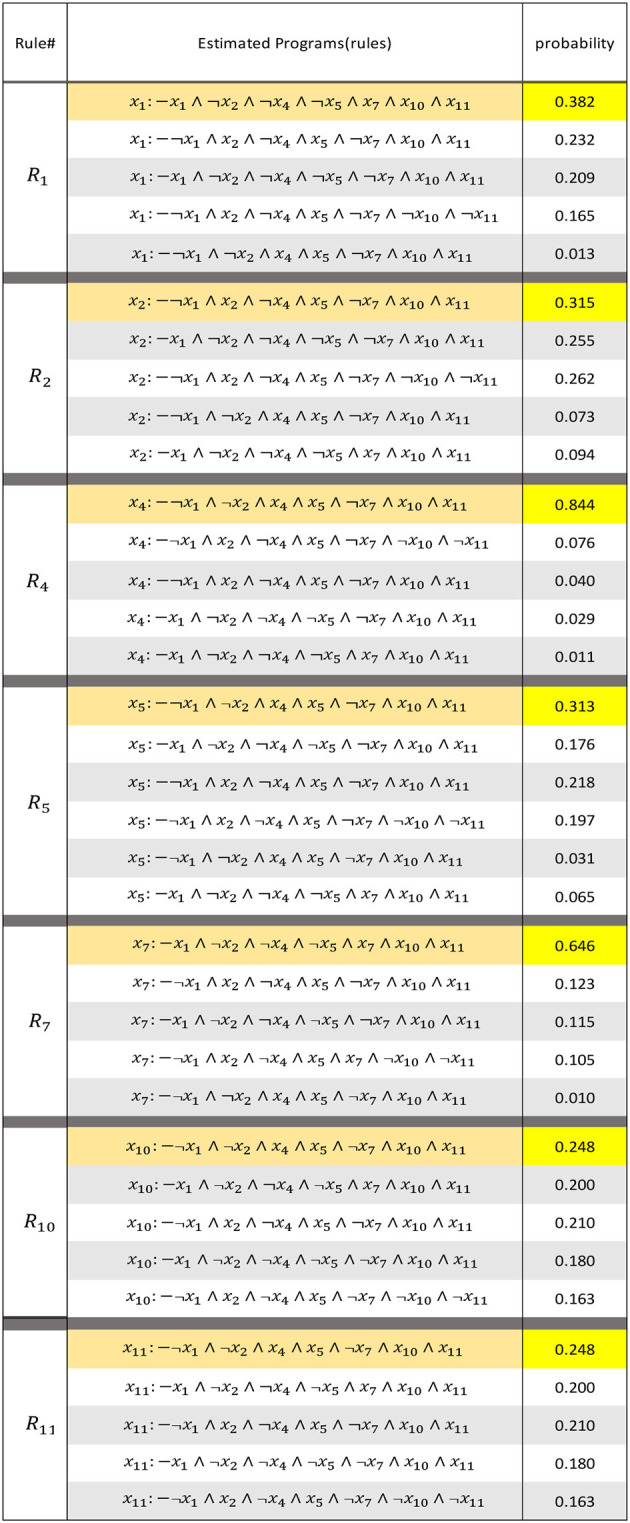

*For each variable, all rules of normal logic programming (NLP) form are listed and most representative rules are highlighted*.

**Figure 8:**
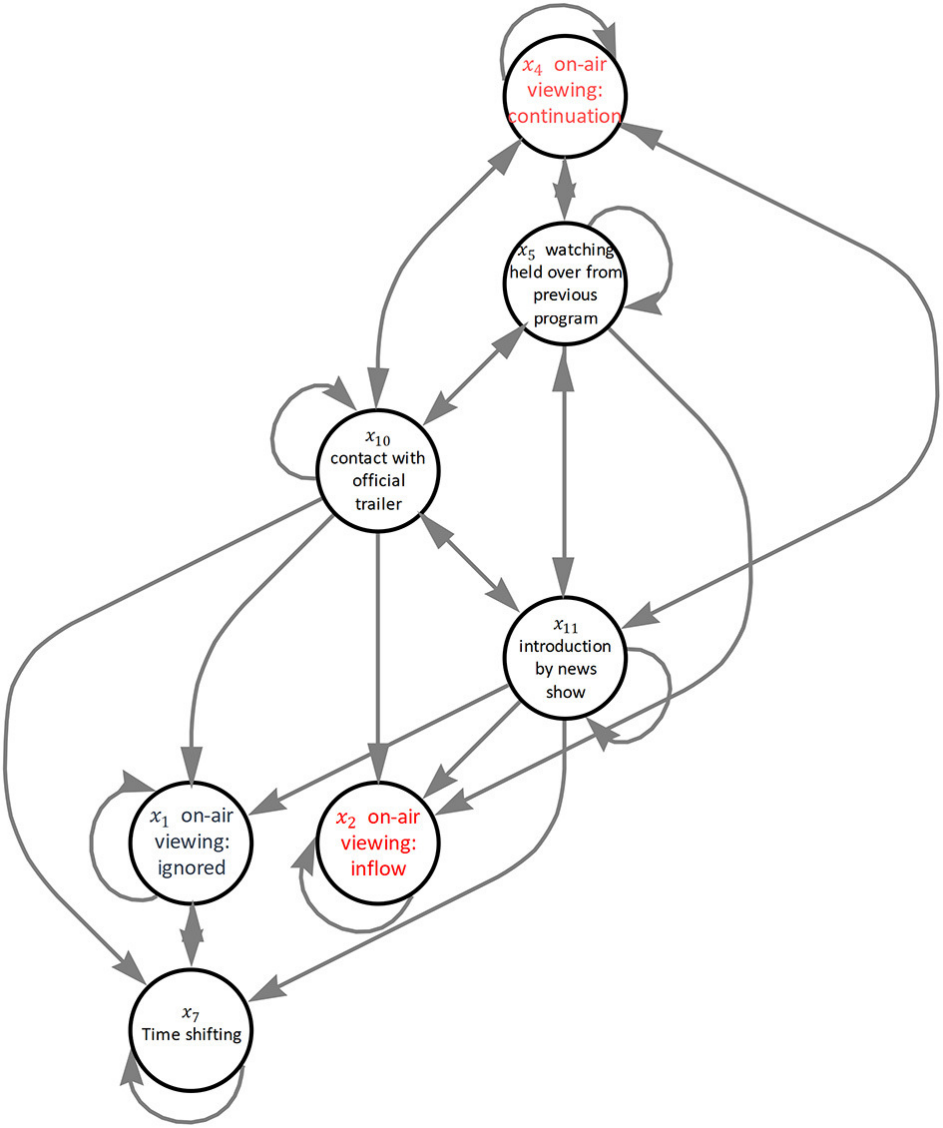
Result of interaction graph. The arrows indicate head-body relationship among the variables.

Figure 8 shows an interaction graph using the top rules at each *R* in the above-described list. We can see how each explanatory variable is connected to the dummy variables *x*_1_, *x*_2_, and *x*_4_, which indicate the viewing dynamics by differences. For instance, *x*_2_ is under the influence of variables *x*_5_, *x*_10_, and *x*_11_. These suggest that in order to increase the inflow of content viewing, the viewer should be met with an attractive hook as they routinely turn on the device.

**Figure 9:**
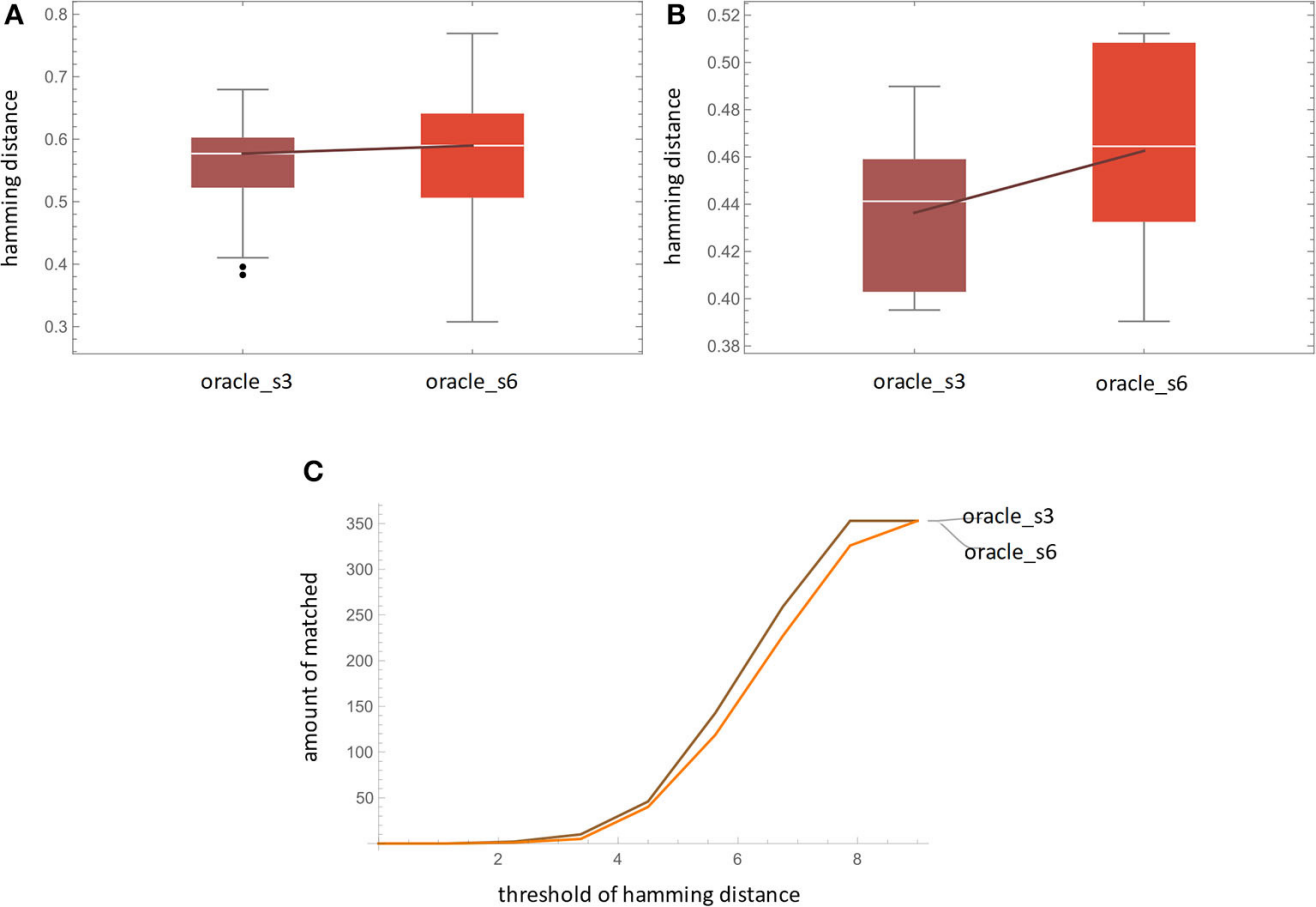
Result of **(A)** accuracy by Hamming distance, **(B)** fidelity, and **(C)** accuracy by matched count.

For measuring accuracy, we use the Hamming distance between the LSTM's prediction **Ŷ** and the ground truth **D**^*u*^. For oracle_s3, each entry contains 6 predictions based on #1-3 logs observed. For oracle_s6, each entry contains 3 predictions based on #1-6 logs observed. For each ${\hat{\text{y}}}_{i}$, we measure its hamming distance corresponding to every kind of pattern in ${\text{x}}_{i}^{u}$. The result (Figure 9A) shows that the difference of the averaged Hamming distances of oracle_s3 and oracle_s6 is not significant, which suggest that both amounts of data are feasible for LSTM's training.

Figure 9B shows the amount of matched prediction. “Matched prediction” means that if the Hamming distance is below a threshold, we consider the prediction $\hat{\text{Y}}$ and its ground-truth **D**^*u*^ is matched. This can be used as a trade-off treatment between noise in real-world data. Given two 13-dimensional binary vectors, the hamming distance is 0 when the vectors are identical and is 13 when they are totally different. The hamming distance we used is normalized by the vector length. From the figure, at all threshold levels, oracle_s3 outperforms oracle_s6, which supports the results of the previous paragraph.

For measuring fidelity, as shown in Figure 9C, we use the Hamming distance between LSTM's prediction $\hat{\text{Y}}$ and its HMM simulation result $\hat{\text{Y}}$. For oracle_s3, data from 6 transitions is used as training data for HMM. Then the trained HMM simulates the emission 9 times and the last 6 patterns are selected as HMM's output. The Hamming distances between $\hat{\text{Y}}$ and **Y** are computed. The result shows that oracle_s3 has a better fidelity than oracle_s6. This may be due to the fact that the former provides more HMM training data, which benefits its learning ability.

### Limitations

However, there are many limitations and unexplored areas in our model fusion. Firstly, optimality of model structures for tasks, and relations between hyperparameter manipulation and explainability in LDA and VAE, remain unknown. Following the above will allow precise and appropriate model fusion for data environment requirements from OTT media services and other specific domains. Also, there is a trade-off between process transparency and analytical sophistication. The reason for limiting the number of features of interest to at most four is the constraint of using a color function to reduce them to a grid representation. Due to cognitive biases, however, there may be limits on the number of options that can be simultaneously presented to a single decision-maker and correctly processed. Also, the data environment was small, centralized, and of high quality. Currently, the centralized environment in many businesses is highly reliable for high-stakes decision-making, regardless of scale. In the future, however, issues of observational granularity and accountability according to format, as well as issues of data fusion, along with ethical and liability issues, will become much more developed.

## Conclusion

**Figure 10:**
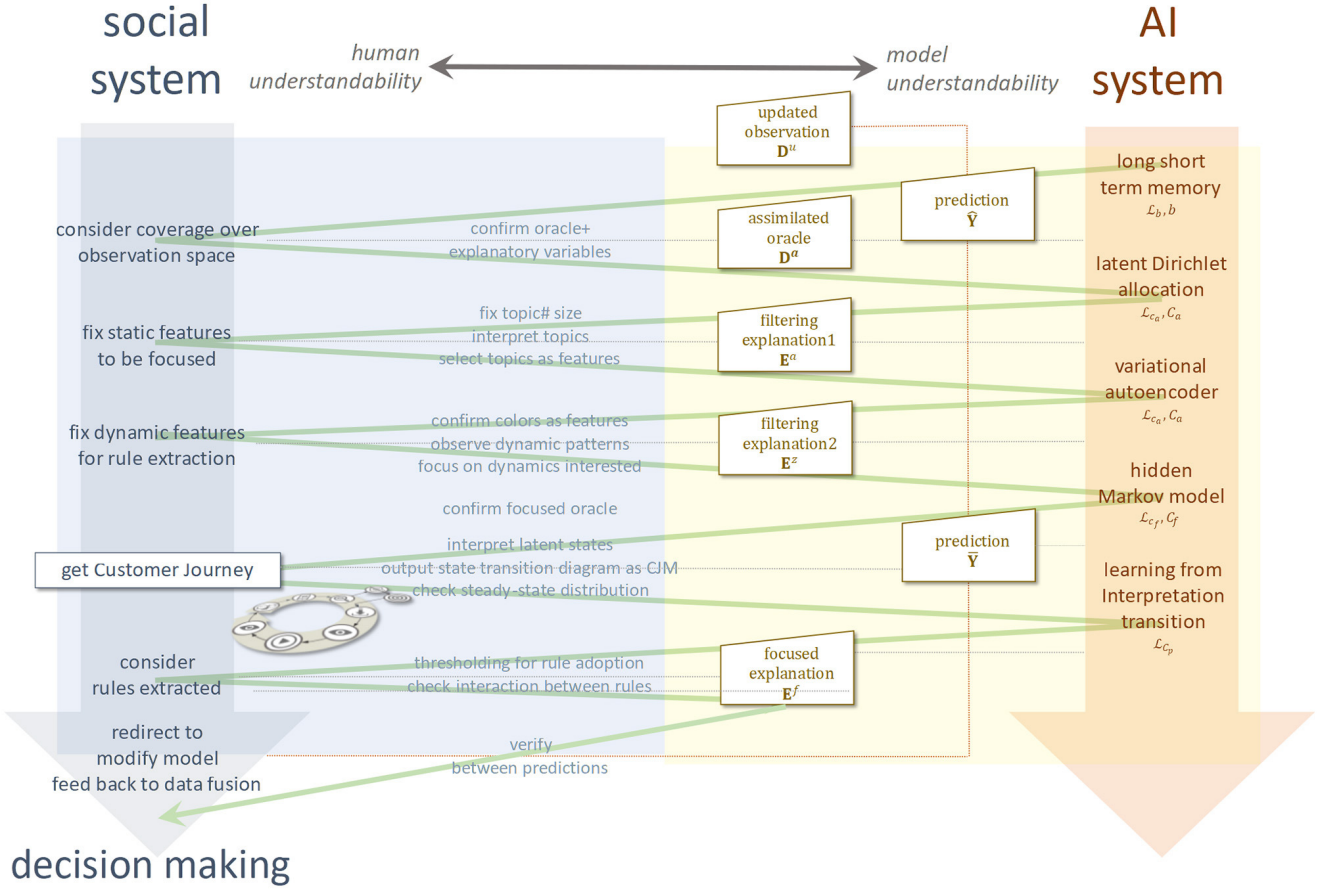
Customer journey mapping based on a human-AI system.

In this paper, we presented an approach to the application of explainable human AI systems for high level decision-making in the marketing domain. The essence of this task is the problem formulation. An AGI system that can perform problem formulation has not been realized yet. To fulfill the gap, we proposed CJM automation through explainable model fusion for addressing the question of how to apply current AI technology to more advanced decision-making. First, we have shown that the customer journey can be regarded as a problem formulation in practical marketing strategy from a management engineering perspective. Next, through explainable alignment, we automated CJM by model fusion, which applies a combination of generative models and ILPs to a process mining frame. Based on the assimilation between the predictions from the black-box deep learning model LSTM and the observed data, we also showed the *post-hoc* explainability through comparison with the above generative HMM. Finally, for experiment, we automatically generated a CJM and extracted the related rules based on dynamic viewing logs of a serial drama in the OTT media service domain. We illustrate the conclusion with Figure 10.

In the traditional broadcasting business, the viewing history has been used only as a material for consideration for the next period after the program ends. This proposal extends the use of the observation environment for strategic data-driven decision-making. In addition, in recent OTT distribution services, the viewing log is input directly into the machine learning of the recommendation system, and the recommendation result is presented to the customer as it is without visualizing the calculation process. The issue was that the programming and production departments were unable to gain knowledge of the planning and production of new content. However, with the introduction of this system, the relationship between the viewer's latent viewing attitude, the content, and the delivery process can be visualized and examined sequentially, making it possible to make more precise and agile service strategy decisions. Thus, our explainable model fusion for CJM has contributed to the explainable human-AI systems for advanced decision-making in local managerial practice. However, this research has only just begun, and we will continue to further optimize model fusion in real-world applications.

## Data Availability Statement

The raw data supporting the conclusions of this article will be made available by the authors, without undue reservation.

## Author Contributions

KO and KI contributed to conception and design of the study. KO organized data processing, performed analysis, and wrote the first draft of the manuscript. Both authors contributed to manuscript revision, read, and approved the submitted version.

## Funding

This research has been supported by Video Research, Ltd. and JSPS KAKENHI Grant Nos. JP17H00763 and JP21H04905. The authors thank Video Research, Ltd. and Every Sync, Inc. for providing valuable actual data on customer behavior history for this research. The funders were not involved in the study design, analysis, interpretation of data, the writing of this article or the decision to submit it for publication.

## Conflict of Interest

KO was employed by SONAR Inc. The remaining author declares that the research was conducted in the absence of any commercial or financial relationships that could be construed as a potential conflict of interest.

## Publisher's Note

All claims expressed in this article are solely those of the authors and do not necessarily represent those of their affiliated organizations, or those of the publisher, the editors and the reviewers. Any product that may be evaluated in this article, or claim that may be made by its manufacturer, is not guaranteed or endorsed by the publisher.
